# Membranome 3.0: Database of single‐pass membrane proteins with AlphaFold models

**DOI:** 10.1002/pro.4318

**Published:** 2022-04-28

**Authors:** Andrei L. Lomize, Kevin A. Schnitzer, Spencer C. Todd, Stanislav Cherepanov, Carlos Outeiral, Charlotte M. Deane, Irina D. Pogozheva

**Affiliations:** ^1^ Department of Medicinal Chemistry College of Pharmacy, University of Michigan Ann Arbor Michigan USA; ^2^ Department of Electrical Engineering and Computer Science College of Engineering, University of Michigan Ann Arbor Michigan USA; ^3^ Department of Biophysics University of Michigan Ann Arbor Michigan USA; ^4^ Department of Statistics University of Oxford UK

**Keywords:** full‐length protein model, network analysis, visualization, web tool

## Abstract

The Membranome database provides comprehensive structural information on single‐pass (i.e., bitopic) membrane proteins from six evolutionarily distant organisms, including protein–protein interactions, complexes, mutations, experimental structures, and models of transmembrane α‐helical dimers. We present a new version of this database, Membranome 3.0, which was significantly updated by revising the set of 5,758 bitopic proteins and incorporating models generated by AlphaFold 2 in the database. The AlphaFold models were parsed into structural domains located at the different membrane sides, modified to exclude low‐confidence unstructured terminal regions and signal sequences, validated through comparison with available experimental structures, and positioned with respect to membrane boundaries. Membranome 3.0 was re‐developed to facilitate visualization and comparative analysis of multiple 3D structures of proteins that belong to a specified family, complex, biological pathway, or membrane type. New tools for advanced search and analysis of proteins, their interactions, complexes, and mutations were included. The database is freely accessible at https://membranome.org.

**Abbreviations**
3D: three‐dimensional
AF2: AlphaFold 2
pLDDT: predicted local distance difference test
RMSD: root‐mean‐square deviation
TM: transmembrane

## 1 INTRODUCTION

Single‐pass (i.e., bitopic) transmembrane (TM) proteins cover up to 50% of mammalian membrane proteins and play key roles in many vital processes, including signal transduction, cell adhesion and communications, immune response, energy conversion, molecular biogenesis and transport, malignant transformation, viral entry, and other cellular processes.^1^ Structural characterization of bitopic proteins is a critical step in understanding the molecular mechanisms of their function and regulation and of the impact of disease‐causing mutations. Unfortunately, determining the three‐dimensional (3D) structure of full‐length bitopic membrane protein remains a challenge for both experimental and computational methods, because these proteins are often composed of multiple structural domains located at different sides of a membrane, and because they exist in many conformational and oligomeric states.

To facilitate structural analysis of bitopic membrane proteins, we previously created the Membranome database,^2^, ^3^ a web resource dedicated to this functionally relevant protein class. The Membranome database compiles available structural data for all bitopic proteins of six organisms (*Homo sapiens*, *Arabidopsis thaliana*, *Dictiostelium discoideum*, *Saccharomyces cerevisiae*, *Escherichia coli*, *Methanococcus jannaschii*) representing the six kingdoms of life. The database provides protein classification, amino acid sequences, membrane topologies, intracellular localizations, domain organization (from UniProtKB^4^ and Pfam^5^), experimentally supported protein–protein interactions, protein complexes, experimental and computed structures of bitopic protein domains, their static pictures produced by PyMOL,^6^ and interactive visualization using 3D viewers. It also includes links to experimental 3D structures of hundreds of water‐soluble domains and dozens of TM domains of bitopic proteins from PDB,^7^ PDBsum,^8^ and OPM.^9^ The original version of the database^2^ contained models of single TM α‐helices predicted and generated by FMAP^10^ for all included bitopic proteins. In 2018, the version 2.0 of the database^3^ was expanded by including more than 2,000 models of energetically stable (average helix association energy was about −6 kcal/mol) TM α‐helical homodimers of bitopic proteins from all six species that were predicted and generated by TMDOCK^11^ and positioned in membranes by PPM.^9^ The Membranome 2.0 database provided a unique point of access to the structural information of bitopic proteins available in 2018.

In the past year, advances in protein structure prediction have powered spectacular progress in structural modeling. At the 14th Critical Assessment of protein Structure Prediction (CASP), the AlphaFold 2 (AF2) system^12^ outperformed all other computational methods, producing models rivaling experimental structures.^12^, ^13^, ^14^, ^15^ Although AlphaFold 2 has a limited applicability in modeling protein dynamics, multiple conformational states, or effects of mutations,^16^, ^17^ the approach is thought to achieve a significant progress in predicting the structure of a single protein chain.^12^ Public availability of the AF2 source code and the recent release of the AlphaFold DataBase^18^ with over half a million protein models, including the full proteomes of 16 model organisms and 32 pathogens, are starting to have a transformative impact on structural biology.^13^, ^15^, ^16^ Despite the varying quality of AF2‐generated models, they have been added to high‐quality authoritative resources for protein sequences, structures, and functional information, such as UniProt^4^ and PDBsum.^19^ The availability of high‐accuracy predictions for a significant portion of many organisms' proteomes is a novel source of information into bitopic proteins.

In this work, we present an upgraded version of the Membranome database that includes AF2 models of bitopic proteins, along with additional information about protein interactions, complexes, and pathways, and new functionalities for protein analysis. Protein models from the AlphaFold DataBase have been modified and validated by positioning them with respect to membrane boundaries. To ensure reliability of new models, they have also been compared to available experimental structures of corresponding proteins. These improvements result in a modern database intended to further the study and understanding of bitopic proteins.

## 2 NEW CONTENT OF MEMBRANOME 3.0

### 2.1 New information for bitopic proteins

A vast sample of new structural data on bitopic proteins was incorporated to the Membranome 3.0 database from various bioinformatics resources, including 9,078 PDB structures of protein domains, 5,664 direct protein–protein interactions, 1,791 validated complexes, and 1,051 mutations in TM domains. Structural and functional information about bitopic proteins and their complexes was filtered to keep the most reliable information supported by publications. For example, only “high‐quality” and “level 2” interactions were taken from HINT^20^ and APID,^21^ respectively. To get additional protein–protein interactions, protein pages were linked to IntAct,^22^ BioGrid,^23^ and STRING.^24^ Bitopic proteins complexes were collected from Reactome,^25^ Complex Portal,^26^ CORUM,^27^ and PDB.^7^ Mutations in TM α‐helices of bitopic proteins were taken from MutHTP.^28^ Biological pathways were compiled from KEGG,^29^ Reactome,^25^ BioCyc,^30^ WikiPathways,^31^ and HMDB^32^ databases and classified into classes and sub‐classes. Models of individual TM α‐helices were recalculated using the latest version of our FMAP program.^10^

### 2.2 AlphaFold models of full‐length monomers positioned in membranes

**FIGURE 1:**
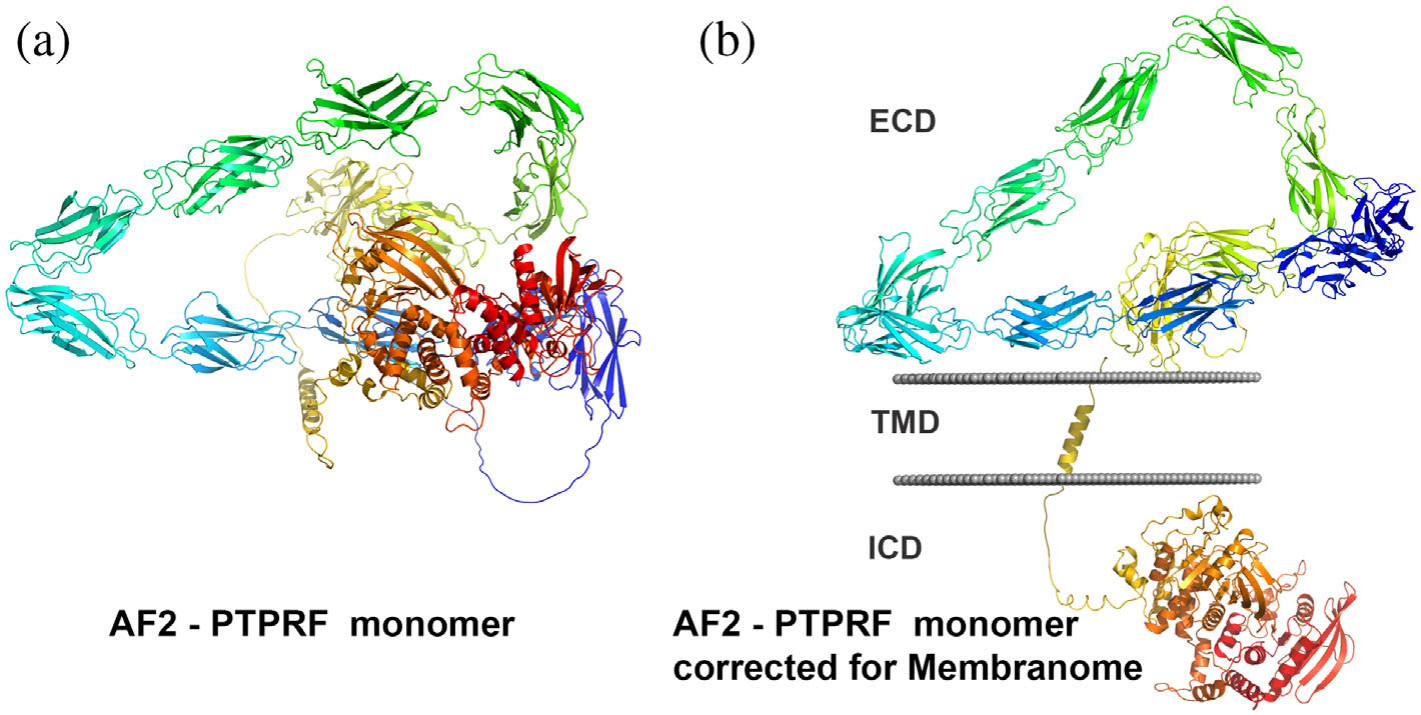
Modeling of full‐length bitopic membrane proteins. (a) AF2‐generated model of receptor tyrosine phosphatase F (PTPRF). (b) AF2 model of PTPRF parsed into the extracellular domain (ECD), TM domain (TMD), and the intracellular domain (ICD) by D‐linker and positioned in membranes by PPM 3.0. Rainbow colored cartoon representations were produced by PyMOL. PPM‐calculated membrane boundaries are shown by gray dots

Despite the success of AlphaFold 2 in accurate predicting single‐chain protein structures, there is a wide variation in model quality between (and within) proteins. A quality assessment score, the predicted local distance difference test (pLDDT) is natively produced by the AF2 system.^13^ High‐confidence predictions (pLDDT > 70, threshold resulting from benchmark on a test set^13^) cover roughly 62% of the human proteome and 92% of sequences in *E. coli*.^33^ Despite progress, there are many proteins and portions of proteins whose AF2 structural models may not be accurate. Models of single‐pass transmembrane proteins are especially problematic, as they include multiple extracellular (EC) and intracellular (IC) domains that can change their relative positions due to the flexibility of connecting loops. One salient problem is that, as AF2 does not consider spatial separation of domains located at opposite membrane sides, it often generates models of large single‐pass TM proteins with intertwined TM, EC, and IC domains (Figure 1a).

The AlphaFold DataBase^18^ contains structural models of almost 99% of the bitopic proteins included in the Membranome database. For each protein with amino acid sequences longer than 2,700 residues, a single model containing TM α‐helix was taken from a set of 1,400‐residue models progressively shifted by 200 residues that are provided by the AlphaFold database. Models of 110 bitopic proteins missing in the AlphaFold database were calculated by AF2 using the standard protocol, code, parameters, and databases available through the CC BY‐NC 4.0 license.^13^

To fix models with incorrectly intertwined domains located at opposite membrane sides, we developed an in‐house program D‐linker that parses EC, IC, and TM parts of a protein, optimizes their spatial positions in membrane using PPM 3.0, and reassembles them at both membrane sides (Figure 1b). The D‐linker also removes N‐terminal signal sequences of bitopic proteins and unstructured regions of low confidence (pLDDT < 70) from N‐ and C‐termini, while keeping all confidently predicted regions (pLDDT ≥ 70) and connecting loops. A significant number of bitopic protein models from the AlphaFold database have distorted or partially unfolded TM α‐helices. To ensure the correct positioning in membrane of these models, their TM segments were automatically superposed by D‐linker with TM α‐helices modeled and oriented in membranes by FMAP.^10^ Several proteins, such as phospholipid scramblases, BCL‐2‐like apopotosis regulators, and chloride intracellular channel proteins from the thioredoxin superfamily, are known to adopt water‐soluble and TM forms. Structural models predicted by AF2 for water‐soluble forms of these proteins were included to Membranome. Ultimately, 5,690 AF2 models were modified, positioned in membrane and included in the Membranome 3.0 database.

To validate these models, we compared them with subunits of 9,093 experimental 3D structures derived from 947 available bitopic proteins representing all considered organisms, except *D. discoideum*. We excluded 105 bitopic proteins with experimental structures containing less than 45 residues. Experimental structures were superimposed with predicted models by TM‐align tool using sequence independent and dependent alignment settings.^34^ The average root‐mean‐square deviation (RMSD) value was less than 2 Å for sets of overlapping residues for single protein domains and semirigid multidomain proteins (Tables 1, S1–S5), but it increased to 2.5–6 Å for multidomain proteins connected by flexible loops or while comparing models with fused protein structures with hybrid amino acid sequences (Table S6). In average, about a half of each AF2 model was covered by experimental structures. These results underscore the high reliability of AF2 method for folded protein regions predicted with high confidence.

**TABLE 1:** Verification of AF2‐generated models by comparison with experimental structures

Bitopic proteins in Membranome 3.0			Superposition of AF2 models with PDB structures^a^				
Species	*N*_prot_ ^b^	*N*_AF2_ ^c^	*N*_AF2_ ^c^	R_over‐AF2_ ^d^	R_over‐AF2_ ^e^ (%)	RMSD^f^ (Å)	Identity^g^ (%)
*H. sapiens*	2,383	2,368	788	271 ± 184	53 ± 22	1.5 ± 0.9	97 ± 10
*A. thaliana*	2,105	2069	52	215 ± 106	51 ± 24	1.0 ± 1.0	98 ± 4
*S. Cerevisiae*	383	374	69	104 ± 21	26 ± 17	1.1 ± 0.5	100 ± 1
*D. discoideum*	605	598	0	0	0	0	0
*E. coli*	205	204	34	476 ± 358	83 ± 10	2.8 ± 1.8	99 ± 1
*M. jannaschii*	77	77	4	131 ± 29	77 ± 9	0.6 ± 0.5	99 ± 1

^a^ Average values with *SD*. One PDB entry with the largest number of overlapped residues was selected for each protein.

^b^ *N*_prot_, number of bitopic proteins.

^c^ *N*_AF2_, number of AF2 models included in Membranome database.

^d^ *R*_over‐AF2_, number of overlapping residues between AF2 model and PDB structure.

^e^ Percentage of overlapping residues between AF2 model and PDB structure.

^f^ RMSD for Cα‐atoms.

^g^ Sequence identity between overlapping residues in AF2 models and PDB structures.

**FIGURE 2:**
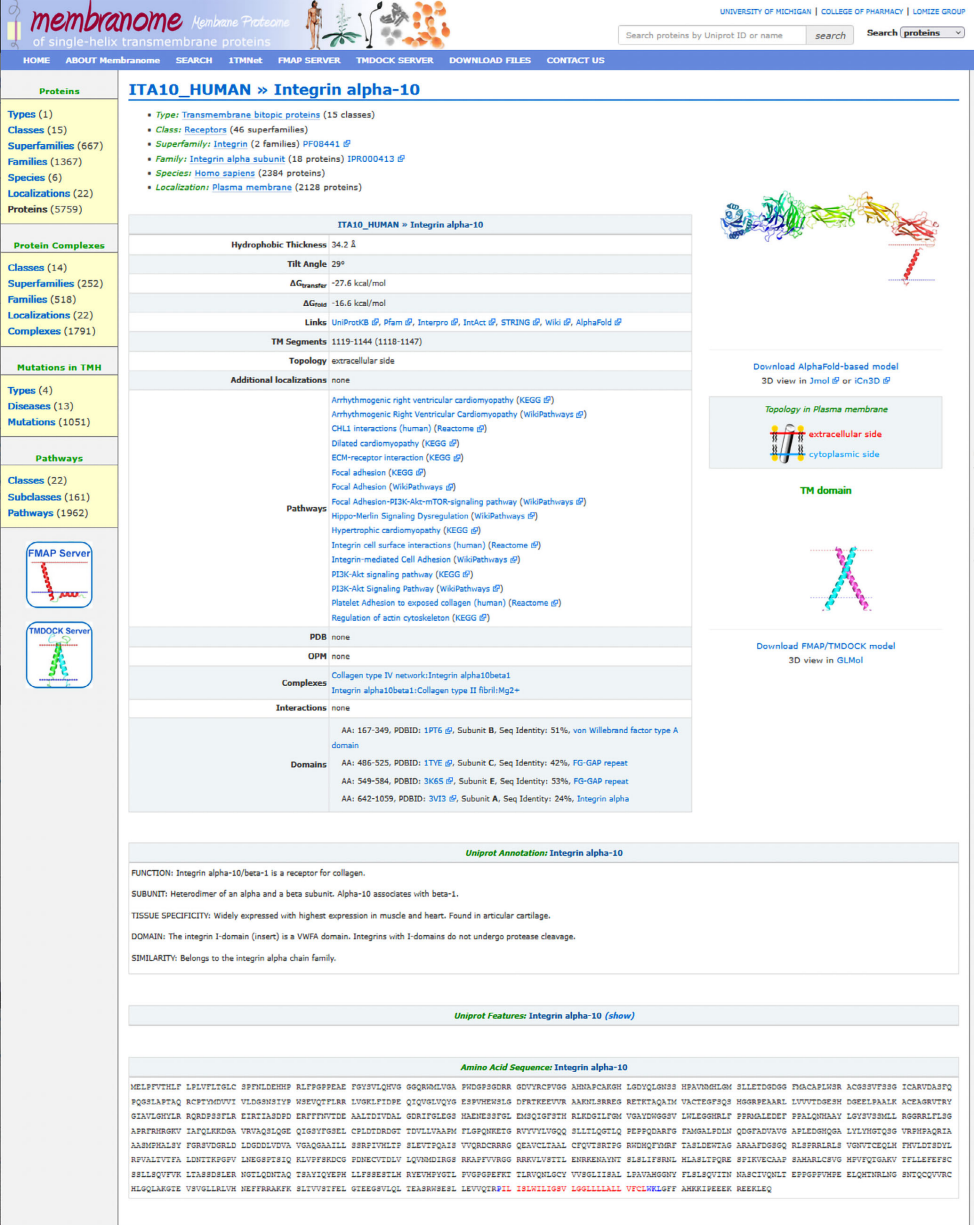
Membranome 3.0 page for integrin alpha‐10 (UniProt ID: ITA10_HUMAN)

The incorporation of AF2‐generated models has several advantages for improving database content and advancing protein analysis. First, these structural models provide atomic representation of the complex domain architecture of full‐length bitopic proteins (Figure 2). Second, comparisons of collections of bitopic membrane proteins in Membranome and the AlphaFold database help refining the sets of all bitopic proteins from six organisms by removing mispredicted polytopic and monotopic proteins from Membranome and by revising and recalculating bitopic proteins that are missing in the AlphaFold DataBase. After corrections, the final bitopic protein sets for *H. sapiens*, *A. thaliana*, *D. discoideum*, *S. serevisiae*, *E. coli*, *M. jannaschii* contain 2,383, 2,105, 605, 383, 205, and 77 entries, respectively (Table 1). Third, comparison of multiple static pictures of protein models during database search or browsing helps to find errors in membrane topology and to revise the structure‐based classification of proteins. The visual comparison of full‐length models of proteins from the same functional category (family, complex, biological pathway, or membrane type) for evolutionarily distant organisms allows exposing their structural features that could be essential for specific protein functions and protein–protein interactions.

The database provides downloadable coordinates only for AF2 models of protein monomers, even though many bitopic proteins are known to form functional dimers or higher order oligomers. Experimental structures of such dimers and multimers available from PDB and OPM databases are linked to Membranome pages for individual bitopic proteins. Besides, Membranome 3.0 includes more than 2,000 downloadable coordinate files of TM α‐helical homodimers that were modeled by TMDOCK.^11^ The full‐length protein dimers could be generated by the AlphaFold‐Multimer (AFM) program.^35^ However, the quality and the reliability of modeling protein dimers by AFM remain rather limited. For a set of 4,433 protein complexes, the AFM successfully predicted dimer interface (DockQ ≥ 0.23) in 67% cases and produced high‐accuracy models (DockQ ≥ 0.8) in 23% cases.^35^ Modeling of dimers of full‐length multidomain bitopic proteins remains a challenging problem. Therefore, we did not include any low‐reliability predictions of full‐length protein dimers in the Membranome 3.0 database.

## 3 NEW FUNCTIONALITIES OF MEMBRANOME 3.0

The new web tool, 1TMnet, was created for analysis of structural and functional (pathways) interaction networks of bitopic proteins (https://membranome.org/1tmnet). For a set of user‐selected proteins from the database, 1TMnet generates interactive tables and graphs that show structural and functional relations between these proteins based on experimentally proven interactions, known complexes, and associations in biological pathways. For example, while selecting *Homo sapiens* and “EGFR” in search boxes (Figure S1), the user gets a table of 29 proteins associated with the human EGFR protein. By selecting all 29 proteins from the table, the user gets graphs and tables for 10 direct (Figure S2) and 42 indirect (Figure S3) interactions between selected bitopic proteins, together with tables presenting 15 complexes with selected proteins and 115 pathways related to these proteins (only complexes and pathways with at least two proteins from the set are included). Direct interactions and protein complexes obtained using 1TMnet facilitates analysis of bitopic protein partners participating in formation of functional protein heteromers. Collecting structural information for proteins from evolutionarily distant organisms in the databases and providing the tools for advanced search, protein network analysis, and interactive 3D visualization facilitates comparative structural and evolutionary analysis of bitopic proteins. To facilitate the interactive visualization of protein structures, iCn3D,^36^ and GLmol^37^ web‐based 3D viewers were included, in addition to JMol.^38^

## 4 DATABASE IMPLEMENTATION

The Membranome database was redeveloped using the Ruby on Rails server‐side web application framework and the PostgreSQL database management system for the back end. The front‐end application was developed using ReactJS. The database is hosted on the Heroku Cloud platform with assets (static protein images and PDB files) stored on the Google Cloud platform. Firebase hosting was used for the front end of the website. The Membranome website also provides access to the FMAP,^10^ TMDOCK,^11^ and 1TMnet web tools. 1TMnet is written in Ruby language on the back end and uses the ActiveRecord library to generate SQL to query the membrane database for results based on the inputs. The front‐end uses the TypeScript programming language and the Cytoscape.js library.^39^

## AUTHOR CONTRIBUTIONS

**Andrei L. Lomize:** Investigation (lead); methodology (lead); software (lead). **Kevin A. Schnitzer:** Software (equal). **Spencer Todd:** Software (equal). **Stanislav Cherepanov:** Formal analysis (equal). **Carlos Outeiral:** Formal analysis (equal). **Charlotte Deane:** Writing – review and editing (equal). **Irina Pogozheva:** Writing – original draft (lead); writing – review and editing (lead).

## CONFLICT OF INTEREST

The authors declare no competing financial interests.

## Supporting information


**Appendix S1** Supporting Information
