# Supplementary material for: Membranome 3.0: Database of single‐pass membrane proteins with AlphaFold models
Source: Protein Sci. 2022 Apr 28;31(5):e4318. doi: 10.1002/pro.4318 (PMC9047035; doi:10.1002/pro.4318)
Supplement: Supplementary file 1 — Appendix S1 Supporting Information [file PRO-31-e4318-s001.pdf]

Supplementary Materials

Andrei L. Lomize[1], Kevin A. Schnitzer[2], Spencer C. Todd[2], Stanislav Cherepanov[3], Carlos Outeiral Rubiera[4], Charlotte M. Deane[4], Irina D. Pogozheva[1]

[1]Department of Medicinal Chemistry, College of Pharmacy, University of Michigan, 428 Church St., Ann Arbor, MI, 48109-1065 USA;

[2]Department of Electrical Engineering and Computer Science, College of Engineering, University of Michigan, 1221 Beal Ave, Ann Arbor, MI 48109-2102;

[3]Department of Biophysics, University of Michigan, 930 N. University Avenue, Ann Arbor, MI, 48109-1065, USA;

[4] Department of Statistics, University of Oxford, 24-29 St Giles', OX1 3PB, United Kingdom.

TABLE of CONTENTS

Page 2 **Figure S1**. 1TMnet input page.

Page 3 **Figure S2**. Direct protein-protein interactions for EGFR_HUMAN produced by 1TMnet.

Page 4 **Figure S3**. Indirect protein-protein interactions for EGFR_HUMAN produced by 1TMnet.

Page 5 **Table 1**. Verification of AF2-generated models of 788 bitopic proteins from *Homo sapiens* by comparison with experimental structures from PDB.

Page 42 **Table 2**. Verification of AF2-generated models of 52 bitopic proteins from *Arabidopsis thaliana* by comparison with experimental structures from PDB.

Page 45 **Table 3**. Verification of AF2-generated models of 69 bitopic proteins from *Saccharomyces cerevisiae* by comparison with experimental structures from PDB.

Page 48 **Table 4**. Verification of AF2-generated models of 34 bitopic proteins from *Escherichia coli* by comparison with experimental structures from PDB.

Page 50 **Table 5.** Verification of AF2-generated models of 4 bitopic proteins from *Methanococcus jannaschii* by comparison with experimental structures from PDB.

Page 50 **Table 6**. AF2-generated models with coordinate R.M.S.D. larger than 5 Å from experimental structures

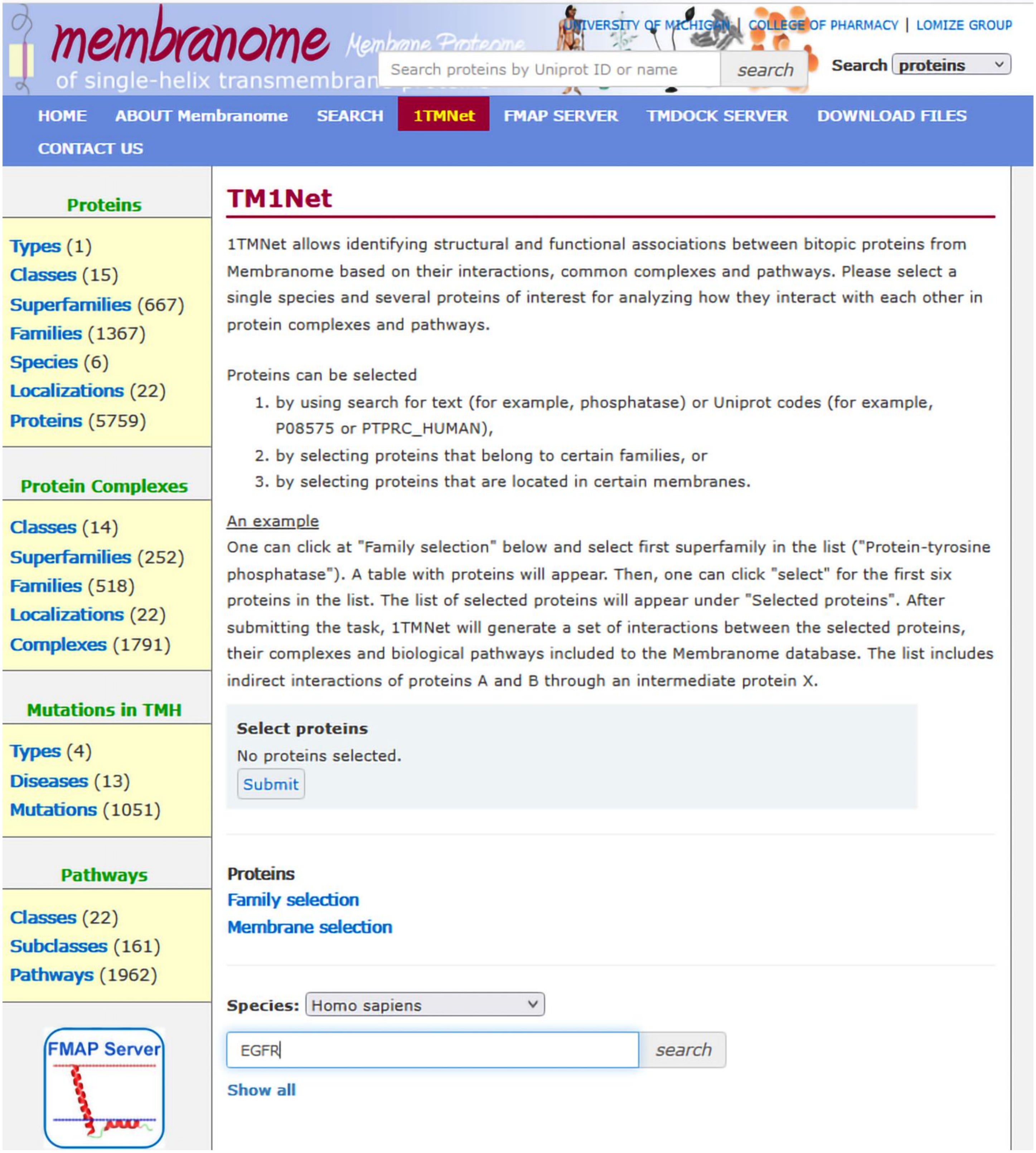

Figure S1. 1TMnet input page.

Direct protein-protein Interactions (10 results)	
**Protein 1**	**Protein 2**
CD44 antigen (CD44_HUMAN)	Receptor tyrosine-protein kinase erbB-2 (ERBB2_HUMAN)
Disintegrin and metalloproteinase domain-containing protein 17 (ADA17_HUMAN)	Protransforming growth factor alpha (TGFA_HUMAN)
Epidermal growth factor receptor (EGFR_HUMAN)	Receptor-type tyrosine-protein phosphatase eta (PTPRJ_HUMAN)
Epidermal growth factor receptor (EGFR_HUMAN)	Receptor tyrosine-protein kinase erbB-4 (ERBB4_HUMAN)
Epidermal growth factor receptor (EGFR_HUMAN)	Mucin-1 (MUC1_HUMAN)
Probetacellulin (BTC_HUMAN)	Receptor tyrosine-protein kinase erbB-2 (ERBB2_HUMAN)
Protransforming growth factor alpha (TGFA_HUMAN)	Receptor tyrosine-protein kinase erbB-2 (ERBB2_HUMAN)
Receptor tyrosine-protein kinase erbB-2 (ERBB2_HUMAN)	Receptor-type tyrosine-protein phosphatase kappa (PTPRK_HUMAN)
Receptor tyrosine-protein kinase erbB-2 (ERBB2_HUMAN)	Receptor-type tyrosine-protein phosphatase eta (PTPRJ_HUMAN)
Receptor tyrosine-protein kinase erbB-2 (ERBB2_HUMAN)	Receptor tyrosine-protein kinase erbB-4 (ERBB4_HUMAN)

Visualization of Direct Protein-Protein Interactions

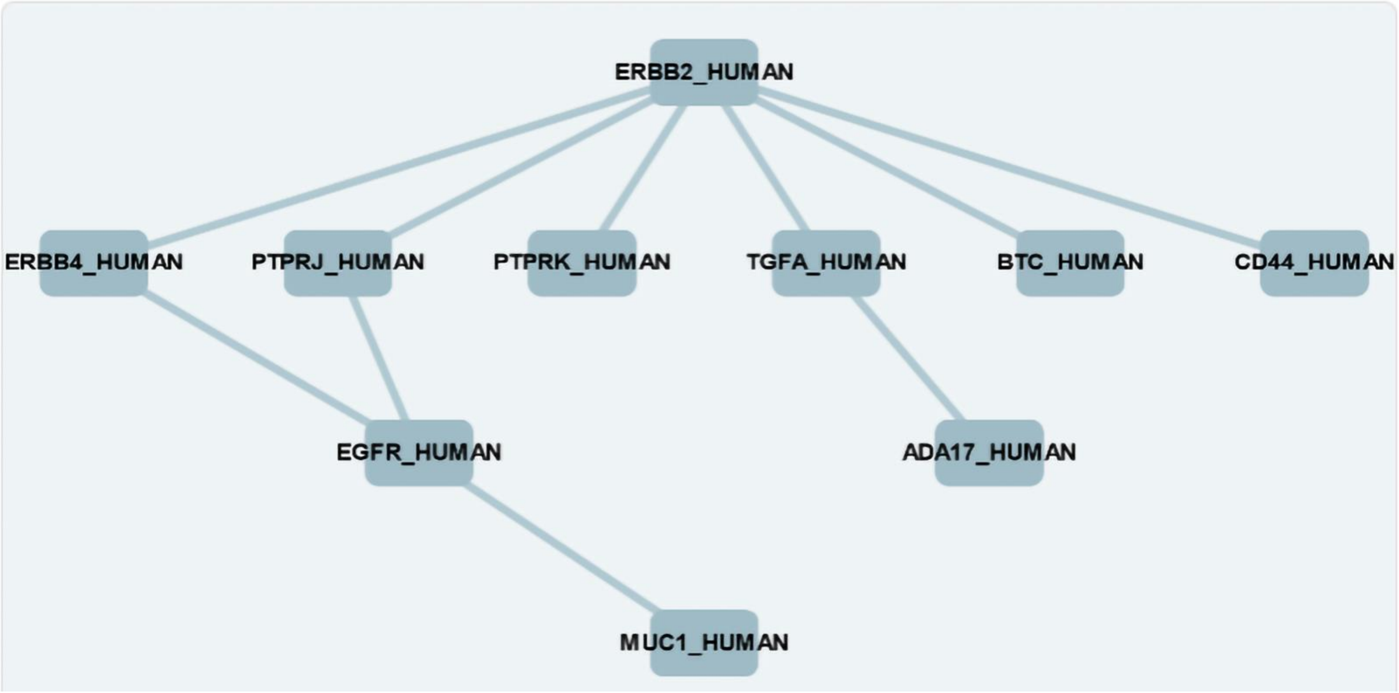

Figure S2. Direct protein-protein interactions for EGFR_HUMAN produced by 1TMnet.

Indirect protein-protein Interactions (42 results)

Protein 1	Through	Protein 2
CD44 antigen (CD44_HUMAN)	4F2 cell-surface antigen heavy chain (4F2_HUMAN)	Epidermal growth factor receptor (EGFR_HUMAN)
CD44 antigen (CD44_HUMAN)	Receptor tyrosine-protein kinase erbB-2 (ERBB2_HUMAN)	Protransforming growth factor alpha (TGFA_HUMAN)
CD44 antigen (CD44_HUMAN)	Receptor tyrosine-protein kinase erbB-2 (ERBB2_HUMAN)	Receptor tyrosine-protein kinase erbB-4 (ERBB4_HUMAN)
CD44 antigen (CD44_HUMAN)	Receptor tyrosine-protein kinase erbB-2 (ERBB2_HUMAN)	Receptor-type tyrosine-protein phosphatase kappa (PTPRK_HUMAN)
CD44 antigen (CD44_HUMAN)	Receptor tyrosine-protein kinase erbB-2 (ERBB2_HUMAN)	Receptor-type tyrosine-protein phosphatase eta (PTPRJ_HUMAN)
CD44 antigen (CD44_HUMAN)	Hepatocyte growth factor receptor (MET_HUMAN)	Receptor-type tyrosine-protein phosphatase eta (PTPRJ_HUMAN)
CD44 antigen (CD44_HUMAN)	Hepatocyte growth factor receptor (MET_HUMAN)	Receptor-type tyrosine-protein phosphatase kappa (PTPRK_HUMAN)
CD44 antigen (CD44_HUMAN)	Hepatocyte growth factor receptor (MET_HUMAN)	Epidermal growth factor receptor (EGFR_HUMAN)
Disintegrin and metalloproteinase domain-containing protein 17 (ADA17_HUMAN)	Integrin beta-1 (ITB1_HUMAN)	Epidermal growth factor receptor (EGFR_HUMAN)
Disintegrin and metalloproteinase domain-containing protein 17 (ADA17_HUMAN)	Protransforming growth factor alpha (TGFA_HUMAN)	Receptor tyrosine-protein kinase erbB-2 (ERBB2_HUMAN)
Epidermal growth factor receptor (EGFR_HUMAN)	Receptor-type tyrosine-protein phosphatase C (PTPRC_HUMAN)	Receptor tyrosine-protein kinase erbB-2 (ERBB2_HUMAN)
Epidermal growth factor receptor (EGFR_HUMAN)	Receptor tyrosine-protein kinase erbB-4 (ERBB4_HUMAN)	Receptor tyrosine-protein kinase erbB-2 (ERBB2_HUMAN)
Epidermal growth factor receptor (EGFR_HUMAN)	Peptidyl-prolyl cis-trans isomerase FKBP8 (FKBP8_HUMAN)	Membrane-associated progesterone receptor component 1 (PGRC1_HUMAN)
Epidermal growth factor receptor (EGFR_HUMAN)	Hepatocyte growth factor receptor (MET_HUMAN)	Receptor-type tyrosine-protein phosphatase kappa (PTPRK_HUMAN)
Epidermal growth factor receptor (EGFR_HUMAN)	Receptor-type tyrosine-protein phosphatase beta (PTPRB_HUMAN)	Receptor tyrosine-protein kinase erbB-2 (ERBB2_HUMAN)
Epidermal growth factor receptor (EGFR_HUMAN)	Receptor-type tyrosine-protein phosphatase eta (PTPRJ_HUMAN)	Receptor tyrosine-protein kinase erbB-2 (ERBB2_HUMAN)
Epidermal growth factor receptor (EGFR_HUMAN)	Receptor-type tyrosine-protein phosphatase R (PTPRR_HUMAN)	Receptor tyrosine-protein kinase erbB-2 (ERBB2_HUMAN)
Epidermal growth factor receptor (EGFR_HUMAN)	Receptor-type tyrosine-protein phosphatase T (PTPRT_HUMAN)	Receptor tyrosine-protein kinase erbB-2 (ERBB2_HUMAN)
Mucin-1 (MUC1_HUMAN)	Epidermal growth factor receptor (EGFR_HUMAN)	Receptor-type tyrosine-protein phosphatase eta (PTPRJ_HUMAN)
Probetacellulin (BTC_HUMAN)	Receptor tyrosine-protein kinase erbB-2 (ERBB2_HUMAN)	CD44 antigen (CD44_HUMAN)
Probetacellulin (BTC_HUMAN)	Receptor tyrosine-protein kinase erbB-2 (ERBB2_HUMAN)	Protransforming growth factor alpha (TGFA_HUMAN)
Probetacellulin (BTC_HUMAN)	Receptor tyrosine-protein kinase erbB-2 (ERBB2_HUMAN)	Receptor tyrosine-protein kinase erbB-4 (ERBB4_HUMAN)
Probetacellulin (BTC_HUMAN)	Receptor tyrosine-protein kinase erbB-2 (ERBB2_HUMAN)	Receptor-type tyrosine-protein phosphatase kappa (PTPRK_HUMAN)
Probetacellulin (BTC_HUMAN)	Receptor tyrosine-protein kinase erbB-2 (ERBB2_HUMAN)	Receptor-type tyrosine-protein phosphatase eta (PTPRJ_HUMAN)
Receptor tyrosine-protein kinase erbB-4 (ERBB4_HUMAN)	Epidermal growth factor receptor (EGFR_HUMAN)	Mucin-1 (MUC1_HUMAN)
Receptor tyrosine-protein kinase erbB-4 (ERBB4_HUMAN)	Epidermal growth factor receptor (EGFR_HUMAN)	Receptor-type tyrosine-protein phosphatase eta (PTPRJ_HUMAN)
Receptor tyrosine-protein kinase erbB-4 (ERBB4_HUMAN)	Receptor tyrosine-protein kinase erbB-2 (ERBB2_HUMAN)	Protransforming growth factor alpha (TGFA_HUMAN)
Receptor tyrosine-protein kinase erbB-4 (ERBB4_HUMAN)	Receptor tyrosine-protein kinase erbB-2 (ERBB2_HUMAN)	Receptor-type tyrosine-protein phosphatase kappa (PTPRK_HUMAN)
Receptor tyrosine-protein kinase erbB-4 (ERBB4_HUMAN)	Receptor tyrosine-protein kinase erbB-2 (ERBB2_HUMAN)	Receptor-type tyrosine-protein phosphatase eta (PTPRJ_HUMAN)
Receptor-type tyrosine-protein phosphatase epsilon (PTPRE_HUMAN)	Platelet-derived growth factor receptor beta (PGFRB_HUMAN)	Receptor-type tyrosine-protein phosphatase kappa (PTPRK_HUMAN)
Receptor-type tyrosine-protein phosphatase epsilon (PTPRE_HUMAN)	Platelet-derived growth factor receptor beta (PGFRB_HUMAN)	Receptor-type tyrosine-protein phosphatase eta (PTPRJ_HUMAN)
Receptor-type tyrosine-protein phosphatase eta (PTPRJ_HUMAN)	Cadherin-2 (CADH2_HUMAN)	Receptor-type tyrosine-protein phosphatase kappa (PTPRK_HUMAN)
Receptor-type tyrosine-protein phosphatase eta (PTPRJ_HUMAN)	Receptor tyrosine-protein kinase erbB-2 (ERBB2_HUMAN)	Protransforming growth factor alpha (TGFA_HUMAN)
Receptor-type tyrosine-protein phosphatase eta (PTPRJ_HUMAN)	Receptor tyrosine-protein kinase erbB-2 (ERBB2_HUMAN)	Receptor-type tyrosine-protein phosphatase kappa (PTPRK_HUMAN)
Receptor-type tyrosine-protein phosphatase eta (PTPRJ_HUMAN)	Growth hormone receptor (GHR_HUMAN)	Receptor-type tyrosine-protein phosphatase kappa (PTPRK_HUMAN)
Receptor-type tyrosine-protein phosphatase eta (PTPRJ_HUMAN)	Insulin receptor (INSR_HUMAN)	Receptor-type tyrosine-protein phosphatase kappa (PTPRK_HUMAN)
Receptor-type tyrosine-protein phosphatase eta (PTPRJ_HUMAN)	Hepatocyte growth factor receptor (MET_HUMAN)	Receptor-type tyrosine-protein phosphatase kappa (PTPRK_HUMAN)
Receptor-type tyrosine-protein phosphatase eta (PTPRJ_HUMAN)	Platelet-derived growth factor receptor beta (PGFRB_HUMAN)	Receptor-type tyrosine-protein phosphatase kappa (PTPRK_HUMAN)
Receptor-type tyrosine-protein phosphatase eta (PTPRJ_HUMAN)	Angiopoietin-1 receptor (TIE2_HUMAN)	Receptor-type tyrosine-protein phosphatase kappa (PTPRK_HUMAN)
Receptor-type tyrosine-protein phosphatase kappa (PTPRK_HUMAN)	Receptor tyrosine-protein kinase erbB-2 (ERBB2_HUMAN)	Protransforming growth factor alpha (TGFA_HUMAN)
Tyrosine-protein phosphatase non-receptor type 2 (PTN2_HUMAN)	Growth hormone receptor (GHR_HUMAN)	Receptor-type tyrosine-protein phosphatase eta (PTPRJ_HUMAN)
Tyrosine-protein phosphatase non-receptor type 2 (PTN2_HUMAN)	Growth hormone receptor (GHR_HUMAN)	Receptor-type tyrosine-protein phosphatase kappa (PTPRK_HUMAN)

Visualization of Indirect Protein-Protein Interactions

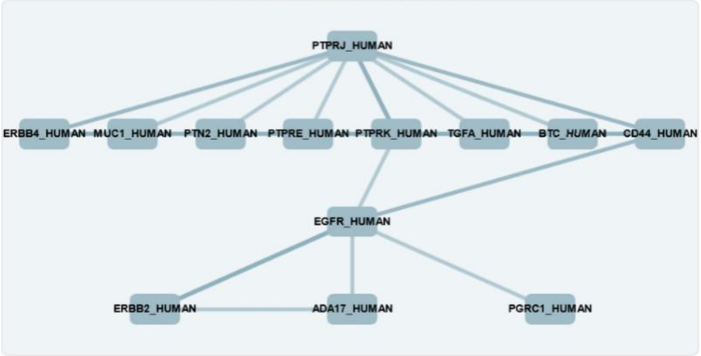

Figure S3. Indirect protein-protein interactions for EGFR_HUMAN produced by 1TMnet.

Table 1. Verification of AF2-generated models of 788 bitopic proteins from *Homo sapiens* by comparison with experimental structures from PDB.

UniProt ID	PDB ID[1]	Chain	N_{over}[2]	Overlapping segments[2]	RMSD, Å [3]	% idenity[4]	Score[5]	%AF2[6]	%PDB[7]
4F2_HUMAN	7CCS	A	469	162-630	2.2	100	93	74	100
A4_HUMAN	3NYL	A	195	370:372-515:517-522:524-567	2.6	97	89	25	99
ABHD6_HUMAN	7OTS	A	292	43:46-335:337	1	99	93	87	100
ACBD5_HUMAN	3FLV	A	92	39-128:130:134	1.4	97	88	17	100
ACE_HUMAN	6EN5	D	613	30-159:163-644:706	1.1	99	94	47	100
ACE2_HUMAN	6M18	B	749	20-729:731-740:743-761:763-771:775	2.3	96	94	93	100
ACVL1_HUMAN	3MY0	N	300	162:195-493	1.7	100	94	60	100
ACVR1_HUMAN	6I1S	A	314	186-193:196:200-280:284-506:510	1.6	99	92	62	98
ADA10_HUMAN	6BE6	C	286	217-458:466-469:641-646:655:658-660:663-669:671-675:706-708:713:715:719-726:749-753	3.6	88	88	38	66
ADA17_HUMAN	2OI0	A	264	216-478:507	1.5	98	87	32	99
ADA22_HUMAN	5Y2Z	K	486	233-717:719	1.1	100	91	54	100
ADA33_HUMAN	1R54	A	205	205:207-410	1.1	99	88	25	100
ADAM8_HUMAN	4DD8	D	208	195-196:198-402:404	1.3	99	86	25	100
AL3A2_HUMAN	4QGK	A	460	2-461	0.5	100	98	95	100
ALK_HUMAN	4FOD	A	308	1083:1086-1136:1143-1277:1279-1280:1285:1288-1405	1.5	98	84	19	100
AMHR2_HUMAN	7L0J	B	105	24-128	1.5	100	80	18	96
AMNLS_HUMAN	6GJE	A	338	20-357	1.6	100	91	75	100
AMPE_HUMAN	4KXD	A	875	76-607:610:612-614:616-954	0.3	99	98	91	100
AMPN_HUMAN	5LHD	C	905	63-967	1.7	100	95	94	100
ANPRC_HUMAN	1JDN	A	407	50-85:92-95:97-463	1.1	100	93	75	100
ANTR1_HUMAN	6CX1	E	180	43-203:209:212-226:245:310-311	1.9	89	93	32	99
ANTR2_HUMAN	7N1O	A	188	77-78:80-82:98:101-102:105-106:109-286	1.7	94	85	38	73
AOC3_HUMAN	4BTY	A	710	53-470:472-763	0.7	100	97	93	100

Table 1. Verification of AF2-generated models of 788 bitopic proteins from *Homo sapiens* by comparison with experimental structures from PDB (continuation).

UniProt ID	PDB ID[1]	Chain	N_{over}[2]	Overlapping segments[2]	RMSD, Å [3]	% idenity[4]	Score[5]	%AF2[6]	%PDB[7]
AOFA_HUMAN	2Z5Y	A	513	12-524	0.4	100	98	97	100
AOFB_HUMAN	6YT2	A	500	2-500:502	0.6	100	97	96	100
APLP1_HUMAN	4RDA	A	201	287-290:295:299-487:489-495	2.5	94	89	31	99
APLP2_HUMAN	5TPT	A	197	373-569	1.7	100	90	26	100
AREG_HUMAN	2RNL	A	46	142-145:147-188	1.8	94	83	18	92
ASAH2_HUMAN	4WGK	B	672	99-638:643-757:763-779	0.3	100	97	86	100
ASB11_HUMAN	4UUC	A	219	66-284	0.7	100	95	68	99
ASGR1_HUMAN	5JQ1	A	130	152-281	0.2	100	97	45	100
ASPH_HUMAN	7E6J	A	429	330-758	0.5	100	96	57	100
ASTRC_HUMAN	6GN5	A	174	322-323:325-341:343-352:354-381:383-499	1.1	99	90	26	100
AVR2A_HUMAN	4ASX	B	306	164:167-171:188-487	1.6	97	92	60	100
AVR2B_HUMAN	2QLU	A	297	188-484	1.2	99	93	58	100
B2CL1_HUMAN	1LXL	A	173	5-32:34-37:41-44:62-71:73-77:112-229:231-232:235-236	3.7	88	82	74	78
B2CL2_HUMAN	1O0L	A	175	18-68:70-81:83-180:182-187:189-191:195-198:200	2.2	88	86	91	93
B2L10_HUMAN	4B4S	A	148	13-116:132-174:179	0.6	97	92	73	100
B3GA1_HUMAN	1V82	A	247	83-156:162-334	0.4	100	96	74	100
B3GA2_HUMAN	2D0J	A	242	78-146:148:152-323	0.8	100	96	75	100
B3GA3_HUMAN	3CU0	A	250	75-140:143:153-335	0.8	99	96	75	100
B3GN2_HUMAN	7JHO	A	331	55-74:76-77:89-397	0.4	99	97	83	100
B4GT1_HUMAN	4L41	C	273	126-398	0.4	99	97	69	100
B4GT7_HUMAN	4IRQ	C	249	79-327	1	99	97	76	100
BACE1_HUMAN	4LC7	A	397	58-220:222-229:231-450:452-457	1.8	96	96	79	99
BACE2_HUMAN	3ZKQ	A	376	75-236:246-385:387-460	0.9	100	95	73	100
BAK_HUMAN	5VWZ	A	166	21-186	1.7	99	86	79	100

Table 1. Verification of AF2-generated models of 788 bitopic proteins from *Homo sapiens* by comparison with experimental structures from PDB (continuation).

UniProt ID	PDB ID[1]	Chain	N_{over}[2]	Overlapping segments[2]	RMSD, Å [3]	% idenity[4]	Score[5]	%AF2[6]	%PDB[7]
BASI_HUMAN	6LZ0	B	198	37:139-150:153-163:165-175:177-185:187:189-202:204-339:341-342:347	3.8	77	93	51	92
BAX_HUMAN	2LR1	A	186	6-13:15-40:42-45:47-194	2.5	95	88	97	97
BCAM_HUMAN	2PF6	A	231	32-261:297	1.4	100	93	37	100
BCL2_HUMAN	2O22	A	153	3-35:97-216	2.5	96	87	64	93
BGAT_HUMAN	6GX2	A	296	59:61-355	0.8	98	97	84	100
BMPR2_HUMAN	3G2F	A	302	197-287:289-364:373:375-396:398-495:497-507:509:511-512	1.2	99	92	29	100
BMR1A_HUMAN	2QJA	D	93	53-54:56-144:146-147	1.5	85	82	17	100
BMR1B_HUMAN	3MDY	A	320	170:175-358:366:369-502	0.9	99	92	64	100
BOC_HUMAN	3N1M	C	104	710-813	0.7	100	76	9	100
BOK_HUMAN	6CKV	A	156	22-177	2.1	97	89	74	100
BST2_HUMAN	3MQB	A	108	50-151:153-158	1.9	96	95	60	99
BT3A1_HUMAN	4F9L	A	214	30-243	0.8	100	93	42	100
BT3A2_HUMAN	4F8Q	A	208	29-181:186-211:213:216-240:242-244	1.9	98	95	62	100
BT3A3_HUMAN	4F8T	A	210	30-212:216-242	1	100	93	36	100
BTC_HUMAN	1IOX	A	49	60-63:66-86:88-91:93-109:112-113:116	3.6	80	85	28	98
BTLA_HUMAN	2AW2	A	110	34-143	1.4	94	92	38	100
C163B_HUMAN	6K0O	A	104	793-896	0.9	98	76	7	100
C2C2L_HUMAN	5TOD	D	153	89-117:119-149:151-176:178-223:234-252:255-256	0.8	99	87	22	100
CABP7_HUMAN	2LV7	A	72	57-128	2.3	100	76	33	72
CAD17_HUMAN	6ULM	B	209	27-235	1.6	100	90	25	100
CAD23_HUMAN	5TFM	A	305	886-918:924-944:948-961:964-1028:1030-1034:1037-1043:1045-1046:1049-1149:1151-1154:1156-1176:1178:1180:1183-1186:1189-1204:1206-1215	2.7	31	84	22	95

Table 1. Verification of AF2-generated models of 788 bitopic proteins from *Homo sapiens* by comparison with experimental structures from PDB (continuation).

UniProt ID	PDB ID[1]	Chain	N_{over}[2]	Overlapping segments[2]	RMSD, Å [3]	% idenity[4]	Score[5]	%AF2[6]	%PDB[7]
CADH1_HUMAN	6CXY	C	213	155-367	1	100	93	24	100
CADH3_HUMAN	6ZTB	I	215	110-323:363	1.6	100	93	26	99
CADH6_HUMAN	5VEB	Y	100	492-591	1.8	100	89	13	93
CADM1_HUMAN	4H5S	B	100	45-143:227	0.8	99	95	23	100
CADM3_HUMAN	1Z9M	B	105	26:28-130:211	0.8	98	96	26	100
CAH12_HUMAN	6RPS	A	262	30-291	0.7	100	98	74	100
CAH14_HUMAN	5CJF	A	267	21-287	0.5	100	97	79	100
CAH4_HUMAN	5KU6	C	264	21-284	0.3	100	97	85	100
CAH9_HUMAN	6Y74	A	258	137-394	0.8	100	97	56	100
CANT1_HUMAN	2H2U	B	317	85-401	0.6	100	98	79	100
CBPD_HUMAN	5AQ0	B	82	382-463	0.6	96	91	6	100
CC90B_HUMAN	6H9M	B	93	61-62:64-100:102-106:108-156	2.4	66	93	37	99
CCD47_HUMAN	6W6L	7	256	152-154:156-176:180-188:194-196:198-202:204-216:218-230:233-242:244-287:296-301:303-341:361-372:374-379:381-436:438-439:499-510:512-513	3.6	68	92	53	88
CD166_HUMAN	5A2F	A	218	28-245	1	100	95	37	100
CD180_HUMAN	3B2D	A	601	26-626	1.4	100	96	91	100
CD19_HUMAN	6AL5	A	232	21-39:48-137:153-176:183:185-278:281-284	1.2	98	85	42	99
CD1A_HUMAN	7KP1	A	274	25-121:125:127-302	1.5	96	94	84	100
CD1B_HUMAN	2H26	A	278	22-299	0.7	100	96	83	98
CD1C_HUMAN	5C9J	A	277	24-299:308	1	91	94	83	99
CD1D_HUMAN	1ZT4	C	278	22-299	1.3	100	95	83	100
CD1E_HUMAN	3S6C	A	271	134-250:254-403:405-408	1.4	98	94	70	73
CD2_HUMAN	1HNF	A	179	27:29-206	1.2	99	95	51	100
CD209_HUMAN	6GHV	C	133	212:253-384	0.7	99	95	33	100
CD22_HUMAN	5VKJ	A	310	21-330	2.5	98	88	37	99

Table 1. Verification of AF2-generated models of 788 bitopic proteins from *Homo sapiens* by comparison with experimental structures from PDB (continuation).

UniProt ID	PDB ID[1]	Chain	N_{over}[2]	Overlapping segments[2]	RMSD, Å [3]	% idenity[4]	Score[5]	%AF2[6]	%PDB[7]
CD226_HUMAN	6ISB	A	220	21-128:130-133:135-242	2.3	99	92	65	100
CD27_HUMAN	5TL5	A	99	23-121	0.9	100	95	38	100
CD28_HUMAN	1YJD	C	118	19-136	0.7	100	93	54	100
CD302_HUMAN	2NAN	A	136	22-81:83-86:88-121:123-127:129-161	2	93	92	59	97
CD320_HUMAN	4ZRQ	D	51	34-36:38-43:71-72:77-116	3	35	75	18	68
CD33_HUMAN	5J06	B	215	18-232	0.9	98	95	59	100
CD38_HUMAN	6VUA	A	255	44:47-300	1.3	98	95	85	100
CD3D_HUMAN	6JXR	d	77	22-98	1.8	100	88	45	71
CD3E_HUMAN	6JXR	f	124	33-156	1.8	100	87	60	100
CD3G_HUMAN	1SY6	A	88	24-28:31-105:113:116:160-165	2.7	85	82	48	52
CD4_HUMAN	3T0E	E	365	26-382:384-390:392	2.6	97	93	80	100
CD40L_HUMAN	1ALY	A	144	113-115:121-214:216-218:220-263	1.5	96	94	55	99
CD44_HUMAN	4PZ4	A	154	18-171	0.9	99	96	21	100
CD5_HUMAN	2JP0	A	107	29:32-137	2.5	98	77	22	97
CD6_HUMAN	5A2E	A	303	43-100:122-366	0.7	99	94	45	100
CD69_HUMAN	3HUP	A	121	79-199	1.2	100	96	61	100
CD79B_HUMAN	3KG5	A	101	45-68:70-72:74-146:148	1.4	96	89	44	98
CD80_HUMAN	1DR9	A	198	34-42:44-149:151-155:157-179:181-186:188-236	3.3	90	95	69	99
CD83_HUMAN	5MJ2	A	81	18-53:56:86-129	1.1	94	90	40	100
CD86_HUMAN	5YXK	A	110	25-134	1	99	96	33	100
CD8A_HUMAN	3QZW	G	114	22-135	0.8	100	94	49	100
CDHR2_HUMAN	5CZR	B	216	20-235	1.1	100	89	16	100
CDHR3_HUMAN	7KNV	A	121	14:18:20:22-134:220-224	2.9	92	84	14	96
CDHR5_HUMAN	6OAE	A	204	26:28-230	1	100	95	24	100
CDON_HUMAN	3N1F	D	102	823-924	0.8	98	77	8	100

Table 1. Verification of AF2-generated models of 788 bitopic proteins from *Homo sapiens* by comparison with experimental structures from PDB (continuation).

UniProt ID	PDB ID[1]	Chain	N_{over}[2]	Overlapping segments[2]	RMSD, Å [3]	% idenity[4]	Score[5]	%AF2[6]	%PDB[7]
CEAM1_HUMAN	6GBG	D	110	34-143	0.5	98	95	21	100
CEAM3_HUMAN	6AW3	A	108	34-141	0.5	99	96	43	100
CGRF1_HUMAN	2EA5	A	56	252-255:279-330	2.3	89	85	17	82
CISD1_HUMAN	6DE9	A	75	30-31:33-105	1.9	97	93	69	100
CISD2_HUMAN	3FNV	B	68	68-135	0.5	98	93	50	100
CLC10_HUMAN	6PY1	A	130	165:180-308	0.6	98	95	41	100
CLC1B_HUMAN	3WWK	C	122	100-221	0.7	100	96	53	100
CLC2A_HUMAN	4IOP	A	117	56-172	0.8	100	96	67	100
CLC2D_HUMAN	4QKH	A	122	68-69:72-191	0.6	98	95	64	99
CLC4A_HUMAN	5B1X	A	130	105-234	0.6	99	97	55	100
CLC4C_HUMAN	4ZET	A	147	67-213	0.8	99	95	69	100
CLC4D_HUMAN	3WHD	C	148	58:61-70:79-215	1.3	99	93	69	100
CLC4E_HUMAN	3WH2	A	131	68:79-173:175-176:178-210	1.1	97	98	60	99
CLC4K_HUMAN	3KQG	A	169	159-327	0.9	99	96	52	100
CLC4M_HUMAN	1SL6	A	168	219-234:245:247-397	0.8	99	89	42	100
CLC5A_HUMAN	2YHF	A	118	70-187	0.5	99	98	63	100
CLC6A_HUMAN	5VYB	A	143	63-70:75-209	0.2	99	97	68	100
CLC9A_HUMAN	3VPP	B	122	111-200:202-203:208-237	0.8	98	93	51	100
CLIC1_HUMAN	4K0G	A	237	5-241	0.9	100	95	98	100
CLIC3_HUMAN	3KJY	A	217	10-51:62-236	0.9	100	96	92	97
CLIC4_HUMAN	2D2Z	B	237	10-14:17-165:174-256	1.4	98	94	94	99
CLIC5_HUMAN	6Y2H	A	223	173-215:218:227-323:327-407:410	1.1	99	93	54	100
CLM1_HUMAN	2NMS	A	112	17:19-128:130	1.2	96	97	39	100
CLM8_HUMAN	2Q87	B	103	22-66:71-93:95-110:112-115:117-131	1.7	96	94	34	97
CMTD1_HUMAN	2AVD	A	219	44-262	0.2	100	98	84	100

Table 1. Verification of AF2-generated models of 788 bitopic proteins from *Homo sapiens* by comparison with experimental structures from PDB (continuation).

UniProt ID	PDB ID[1]	Chain	N_{over}[2]	Overlapping segments[2]	RMSD, Å [3]	% idenity[4]	Score[5]	%AF2[6]	%PDB[7]
CNTP2_HUMAN	5Y4M	A	150	34-183	0.4	98	95	11	100
COL12_HUMAN	2OX8	C	129	606-734	0.9	99	91	17	100
COMT_HUMAN	4XUE	B	215	51-265	0.8	100	97	79	100
COQ8A_HUMAN	4PED	A	372	255:257:260-279:281-308:310-311:314-348:350-356:358-375:377-380:382:384-484:486-543:545-554:556-588:590-612:614-631:633-644	2.5	97	86	58	100
COX41_HUMAN	5Z62	D	144	26-169	0.9	100	96	85	100
COX6C_HUMAN	5Z62	I	73	3-75	1.6	100	96	97	100
COX7B_HUMAN	5Z62	K	49	30-78	0.9	100	97	61	100
COX7C_HUMAN	5Z62	L	47	15:18-63	0.7	98	95	75	100
CP17A_HUMAN	5IRV	C	470	16:20:37-39:42-43:45-226:229-507:509-510	2.3	98	93	93	99
CP19A_HUMAN	5JL9	A	452	45-496	0.5	100	96	90	100
CP1A1_HUMAN	6UDL	B	477	36-512	0.4	100	97	93	100
CP1A2_HUMAN	2HI4	A	480	34-513	0.4	99	97	93	100
CP1B1_HUMAN	6IQ5	A	459	68-307:312-526:528-530:532	1	99	95	85	100
CP21A_HUMAN	4Y8W	C	442	28-265:274-324:332-484	0.5	100	96	89	100
CP2A6_HUMAN	4EJJ	A	465	30-494	0.4	100	98	94	100
CP2AD_HUMAN	4EJG	D	465	30-494	0.6	100	97	94	100
CP2B6_HUMAN	5WBG	B	464	27-273:275-491	0.9	98	96	94	100
CP2C8_HUMAN	2VN0	A	463	28-490	1.2	100	93	94	100
CP2C9_HUMAN	4NZ2	A	464	27-490	1.4	98	94	95	100
CP2CJ_HUMAN	4GQS	A	461	29-136:139-491	1.6	100	95	94	99
CP2D6_HUMAN	5TFT	A	467	31-497	0.5	99	95	94	100
CP2E1_HUMAN	3T3Z	A	463	31-493	0.3	100	97	94	100
CP2R1_HUMAN	3DL9	A	464	38-501	0.3	100	98	93	100

Table 1. Verification of AF2-generated models of 788 bitopic proteins from *Homo sapiens* by comparison with experimental structures from PDB (continuation).

UniProt ID	PDB ID[1]	Chain	N_{over}[2]	Overlapping segments[2]	RMSD, Å [3]	% idenity[4]	Score[5]	%AF2[6]	%PDB[7]
CP3A4_HUMAN	5TE8	B	468	26-211:213-220:222-282:284-285:289-290:292-500	2.3	98	94	93	99
CP3A5_HUMAN	5VEU	C	469	27-495	0.6	100	95	93	100
CP3A7_HUMAN	7MK8	B	462	25-200:204-214:216-218:220:222-244:246:249-268:270-271:273-282:284-285:287:289-500	1.7	90	94	92	99
CP46A_HUMAN	4J14	A	436	56-491	1	100	97	87	100
CP51A_HUMAN	3JUV	A	446	55-235:237-238:240-249:252-504	1.6	98	95	89	100
CP7A1_HUMAN	3V8D	A	476	24-37:39:41-48:51-472:475:477-506	1.2	97	96	94	99
CR1_HUMAN	5FO9	F	193	491-683	2.1	98	71	9	100
CR2_HUMAN	1GHQ	C	134	20-152:200	1	98	83	13	100
CRLF2_HUMAN	5J11	C	190	29-218	0.4	100	95	51	100
CRTAM_HUMAN	3RBG	D	100	24-123	1.5	96	94	25	97
CSF1_HUMAN	5LXF	B	149	32:35-182	1.2	98	92	27	100
CSF1R_HUMAN	4LIQ	E	469	20-88:94-143:145-146:148-150:152-344:346-351:353-356:359-500	1.2	97	91	48	100
CSF2R_HUMAN	4RS1	B	267	35-74:78-100:102:105-229:232-260:266:268-315	0.8	98	94	67	100
CSF3R_HUMAN	2D9Q	B	299	26-237:245-331	1.5	99	88	36	100
CSMD1_HUMAN	2EHF	A	70	13:80:689-691:736-797:846-848	2.7	33	75	5	96
CSPG4_HUMAN	7ML7	B	137	409:411-412:414-477:479-488:490-549	1.5	90	77	6	99
CTLA4_HUMAN	3OSK	B	123	38-156:158-159:161-162	1.4	98	91	55	100
CX6A1_HUMAN	5Z62	G	73	33:35-58:60-63:66-68:70-110	1.4	90	94	67	97
CX7A2_HUMAN	5Z62	J	56	24:26-80	1.1	98	96	67	100
CXAR_HUMAN	3J6N	K	209	24:26-233	1.2	90	94	57	100
CYB5_HUMAN	2I96	A	85	11-94:96	1.9	99	91	63	79
CYB5B_HUMAN	3NER	A	85	27-111	1.2	100	93	57	94

Table 1. Verification of AF2-generated models of 788 bitopic proteins from *Homo sapiens* by comparison with experimental structures from PDB (continuation).

UniProt ID	PDB ID[1]	Chain	N_{over}[2]	Overlapping segments[2]	RMSD, Å [3]	% idenity[4]	Score[5]	%AF2[6]	%PDB[7]
DAF_HUMAN	1OK9	A	254	33-285:287	1.5	99	96	67	100
DAG1_HUMAN	5LLK	A	226	61-162:181-304	0.5	100	90	25	100
DCC_HUMAN	4URT	B	204	844-870:872-876:878-888:890-902:904-1049:1051-1052	1.9	89	81	14	99
DDR1_HUMAN	4AG4	A	340	30-369	0.6	99	93	37	100
DDR2_HUMAN	6FER	D	280	553-582:602-851	1.6	100	87	33	100
DHI1_HUMAN	4IJU	B	271	32-302	1.1	98	96	93	96
DJB12_HUMAN	2CTP	A	73	111-180:182-184	2	89	86	19	94
DJC30_HUMAN	2YUA	A	91	40-124:126-131	2.2	90	82	40	92
DLL1_HUMAN	4XBM	B	401	22-95:97-99:106-131:133-134:136:139:142-430:432:439-440:444:463	2.9	98	90	55	100
DLL4_HUMAN	5MVX	A	251	26-44:49-53:56-57:59-66:68-71:73-74:76-91:94-132:134-145:147-159:161-191:193-199:201-267:269-274:276-287:289-293:296:299-300	4.7	55	94	37	84
DMA_HUMAN	2BC4	C	193	38-230	1.2	98	96	74	99
DMB_HUMAN	2BC4	D	196	21-212:214-216:218	2	98	94	75	99
DMPK_HUMAN	2VD5	A	383	11-300:305-357:360-364:370-388:393:395-409	1.5	98	92	61	98
DNJC1_HUMAN	2CQR	A	65	168-169:171-172:175:189:192:497-500:502-553:556-557	3.2	74	88	12	89
DOA_HUMAN	4I0P	C	180	27-76:78-83:85-208	1.4	97	94	72	99
DOB_HUMAN	4I0P	D	188	29:31-217	2.3	100	96	69	100
DOPO_HUMAN	4ZEL	A	550	45:47-108:110-592:606-609	1.3	99	93	89	100
DPA1_HUMAN	4P4K	E	180	34-213	1.3	100	96	69	99
DPB1_HUMAN	4P5M	B	186	45-46:48-231	2.5	97	93	72	93
DPP10_HUMAN	4WJL	B	719	64-65:67-783	0.7	100	94	90	100
DPP4_HUMAN	5KBY	B	732	36-767	0.8	100	98	96	100

Table 1. Verification of AF2-generated models of 788 bitopic proteins from *Homo sapiens* by comparison with experimental structures from PDB (continuation).

UniProt ID	PDB ID[1]	Chain	N_{over}[2]	Overlapping segments[2]	RMSD, Å [3]	% idenity[4]	Score[5]	%AF2[6]	%PDB[7]
DPP6_HUMAN	1XFD	A	723	127-849	0.9	100	94	84	100
DQA1_HUMAN	1JK8	A	180	27-76:78-207	1.3	91	94	71	99
DQB1_HUMAN	1JK8	B	189	35-138:140-142:144-225	1.8	95	94	72	99
DRA_HUMAN	4I5B	D	184	27-210	1.5	100	95	72	98
DRB1_HUMAN	5NIG	B	195	30-220:223-226	0.9	89	95	73	99
DRB3_HUMAN	2Q6W	B	188	32-219	0.9	100	96	71	100
DRB5_HUMAN	1H15	B	189	30-32:34-219	1.7	98	95	71	100
DSC1_HUMAN	5IRY	A	539	135-673	2.7	100	91	60	100
DSC2_HUMAN	5J5J	A	482	135:137-333:335-468:470-485:488-534:536-546:548-559:563-579:581-589:617-626:652-660:716:723-724:726-729:732:738-741:755-759:762-763	4.3	43	92	54	87
DSCAM_HUMAN	6ZR7	AAA	273	516:595-620:622-633:635-661:663-676:678-694:700-707:710-718:720-784:786-793:795-800:802-808:813-825:827-834:836-840:842-866:872-883:886-890:892-896	4.9	63	81	14	93
DSCL1_HUMAN	1VA9	A	111	728:962:987:989-1095:1180	2.2	96	82	5	91
DSG2_HUMAN	5ERD	A	551	50-526:528-536:539-552:555-565:567-606	3.4	94	93	49	99
DSG3_HUMAN	5EQX	A	438	50-448:450-455:457-488:520	3.2	98	92	44	100
DYSF_HUMAN	7KRB	A	128	8-114:116-119:121-137	2.3	98	85	6	93
E2AK3_HUMAN	4X7L	A	260	585-663:881-957:982-983:986-1087	1.7	98	87	23	100
E41L3_HUMAN	6IBE	A	284	107-389:413	1.2	100	93	26	100
ECE1_HUMAN	3DWB	A	660	101-421:431:433-770	0.9	100	97	86	100
EDA_HUMAN	1RJ7	E	149	241-244:246-390	1.1	97	93	38	100
EFNB1_HUMAN	6THG	F	137	31-164:166-168	1.4	98	93	40	100
EFNB2_HUMAN	6PDL	D	139	27-92:95-167	1.9	100	93	42	100

Table 1. Verification of AF2-generated models of 788 bitopic proteins from *Homo sapiens* by comparison with experimental structures from PDB (continuation).

UniProt ID	PDB ID[1]	Chain	N_{over}[2]	Overlapping segments[2]	RMSD, Å [3]	% idenity[4]	Score[5]	%AF2[6]	%PDB[7]
EFNB3_HUMAN	4BKF	C	140	28:30-118:120-122:124-170	1.2	96	92	41	99
EGF_HUMAN	2KV4	A	46	971-978:981:984-1019:1025	3.7	96	68	4	87
EGFR_HUMAN	3QWQ	A	613	25:27-638	1.3	100	92	51	100
EGLN_HUMAN	5HZW	A	319	200-201:204:354:359-368:370-371:373-434:436:438:441-679	2	95	87	48	48
EMC1_HUMAN	6WW7	A	927	23-24:26-341:367-500:502-503:519-521:523:525-993	2.1	99	91	93	100
EMC10_HUMAN	6Z3W	I	156	50-114:116-120:123-126:129-133:135-178:180-193:196-200:204-216:218	2.7	72	92	60	95
EMC7_HUMAN	6WW7	G	111	43-152:154	1.5	99	90	46	100
EMD_HUMAN	2ODG	C	46	3-48	0.7	100	91	18	98
EMRE_HUMAN	6K7Y	F	54	48-100:102	1.3	98	85	50	100
ENPP1_HUMAN	6WEW	AbA	817	105-921	0.4	100	97	88	100
ENPP3_HUMAN	6C01	A	819	53-871	0.3	100	98	94	100
ENPP4_HUMAN	4LR2	A	379	24-402	0.2	100	98	84	100
ENPP5_HUMAN	5VEM	B	383	19-401	1.2	98	96	80	100
ENPP7_HUMAN	5UDY	A	388	30-417	0.2	100	98	85	100
ENTK_HUMAN	6ZOV	B	234	787-1020	0.8	97	87	23	100
EPCAM_HUMAN	4MZV	A	239	25-263	1.7	98	91	76	99
EPCR_HUMAN	3JTC	B	173	25-197	0.4	100	96	73	100
EPHA1_HUMAN	3KKA	A	64	911-974	1	100	77	7	100
EPHA2_HUMAN	3FL7	A	481	25-58:61:63-108:111:114-153:160-238:241-380:382-464:466:468-477:479-487:489-500:502-513:517-526:529-531	2.8	90	90	49	100
EPHA3_HUMAN	3FXX	A	285	606-772:777:779:784-892:894-895:897:899:903:906-907	1.4	98	87	29	99
EPHA4_HUMAN	4M4R	A	513	27-539	2.3	100	91	52	99
EPHA5_HUMAN	2R2P	A	272	661:663-824:843-951	1.5	97	88	26	96
EPHA7_HUMAN	2REI	A	277	607-779:799-900:902:907	0.9	99	89	28	100

Table 1. Verification of AF2-generated models of 788 bitopic proteins from *Homo sapiens* by comparison with experimental structures from PDB (continuation).

UniProt ID	PDB ID[1]	Chain	N_{over}[2]	Overlapping segments[2]	RMSD, Å [3]	% idenity[4]	Score[5]	%AF2[6]	%PDB[7]
EPHA8_HUMAN	3KUL	B	271	627-792:794-795:797-899	1.3	100	85	27	100
EPHB1_HUMAN	5MJB	A	270	605:608-609:611-646:648-767:787-897	1.2	98	90	27	98
EPHB2_HUMAN	3ZFM	A	248	615-627:632-655:660-765:789-891:893-894	0.6	99	90	24	100
EPHB3_HUMAN	5L6P	A	260	632-778:807-919	1.3	100	87	26	95
EPHB4_HUMAN	6FNK	A	274	600-601:604:607-760:762-766:778-889	1.1	96	89	28	100
EPOR_HUMAN	6MOJ	B	219	28:31-248	1.9	98	90	43	100
ERAP1_HUMAN	6RQX	A	862	46-485:515-554:557-936:939-940	0.6	100	97	92	100
ERBB2_HUMAN	6J71	A	617	22-277:279-284:286-638:644-645	1.1	99	90	49	100
ERBB3_HUMAN	4LEO	C	593	27-38:41:43-112:114-128:130-144:146-155:157:160-171:174:177-187:189:191-204:207-214:217-638	3.8	91	92	44	99
ERBB4_HUMAN	2AHX	B	615	25-639	2	100	91	47	100
EREG_HUMAN	5WB7	E	47	64-109:111	1.4	98	81	28	100
ERN1_HUMAN	6XDB	A	404	561-964	1.2	100	91	41	100
ESYT2_HUMAN	4P42	B	431	192-255:257:259-260:263-371:379-397:414-463:466-524:532-658	1.8	99	90	47	100
FBCD1_HUMAN	4M7F	A	220	239-458	0.3	100	98	48	100
FCAR_HUMAN	1OVZ	A	193	27-219	1.4	98	92	67	100
FCER2_HUMAN	4GKO	L	136	158-293	1.1	100	92	42	100
FCERA_HUMAN	1J86	B	173	26-197:199	0.9	99	93	67	100
FCG2A_HUMAN	1H9V	A	172	37-208	1.3	99	95	54	100
FCG2B_HUMAN	2FCB	A	173	46-218	1.6	99	95	56	100
FCG2C_HUMAN	3WJL	C	160	48-72:81-215	0.5	100	97	50	100
FCG3A_HUMAN	5MN2	A	172	22-191:193-194	2.6	99	97	68	100
FCGR1_HUMAN	4X4M	E	259	21-219:223-282	1.9	94	94	69	100
FCGRN_HUMAN	6QIP	B	267	26-291:293	0.6	100	95	73	100

Table 1. Verification of AF2-generated models of 788 bitopic proteins from *Homo sapiens* by comparison with experimental structures from PDB (continuation).

UniProt ID	PDB ID[1]	Chain	N_{over}[2]	Overlapping segments[2]	RMSD, Å [3]	% idenity[4]	Score[5]	%AF2[6]	%PDB[7]
FDFT_HUMAN	3WEK	A	335	35-368:370	0.9	99	96	80	100
FGFR1_HUMAN	4RWK	A	300	457-646:655-660:663:670-770:772-773	2.2	96	87	36	97
FGFR2_HUMAN	4J96	B	305	446-447:464-766	1.7	99	87	37	100
FGFR3_HUMAN	6PNX	B	294	442-443:457-575:581:585-755:757	1.9	98	87	36	100
FGFR4_HUMAN	4TYG	A	284	438-442:445:447:451-567:583-635:645-648:658-760	2.1	97	87	35	97
FICD_HUMAN	6I7J	A	337	100:103:105-432:434-440	0.7	97	96	74	100
FIS1_HUMAN	1PC2	A	129	5-6:9-125:128:136:138-143:147-148	2.7	94	83	85	85
FKBP8_HUMAN	5MGX	G	266	91-356	0.7	100	96	65	100
FKRP_HUMAN	6KAM	A	446	46-491	0.4	100	97	90	100
FLRT3_HUMAN	5CMP	A	324	29-349:351:353-354	0.6	99	96	50	100
FLT3_HUMAN	3QS9	E	403	79-95:98-101:103-111:113-116:118-119:121:123:125:127-147:149:151-162:164:169-201:215-347:350-436:438-447:452-470:476-481:484-489:496-507:509-531	2.8	89	84	41	100
FLT3L_HUMAN	7NBI	B	135	28-29:31-163	1.6	98	96	57	98
FND3A_HUMAN	1WK0	A	119	257-296:298-300:302-375:453-454	2	93	78	10	87
FNDC5_HUMAN	4LSD	F	97	33-75:77-129:132	1	99	94	46	100
FOLH1_HUMAN	6SKH	A	696	52:56-750	0.6	100	97	93	100
FURIN_HUMAN	5JMO	B	470	108-576:579	0.8	99	96	59	100
FUT8_HUMAN	6VLD	B	469	107-575	0.4	100	97	82	100
G6B_HUMAN	6R0X	E	99	20-36:38-39:41-51:54-68:70:72-76:78-79:81:83-88:91-124:126:128-131	1.4	66	91	41	100
GALT2_HUMAN	4D0T	A	496	75-569:571	1.3	100	96	87	100
GALT4_HUMAN	6H0B	B	521	58-578	1.2	100	96	90	100
GALT7_HUMAN	6IWR	A	546	112-657	1	100	97	83	100

Table 1. Verification of AF2-generated models of 788 bitopic proteins from *Homo sapiens* by comparison with experimental structures from PDB (continuation).

UniProt ID	PDB ID[1]	Chain	N_{over}[2]	Overlapping segments[2]	RMSD, Å [3]	% idenity[4]	Score[5]	%AF2[6]	%PDB[7]
GDAP1_HUMAN	7AIA	AAA	234	22:24-58:60-62:86-171:173-174:177:222-326:328	2.1	97	95	65	90
GFRAL_HUMAN	6WMW	B	192	129-320	0.6	100	92	49	100
GGT1_HUMAN	4GG2	A	343	33-373:439-440	0.9	99	97	60	100
GHR_HUMAN	1HWH	B	196	50-70:81-255	0.9	100	92	31	100
GIMA1_HUMAN	3V70	A	217	26-53:63-251	0.4	100	96	71	100
GIMA4_HUMAN	3LXX	A	165	29-58:66-89:91:96-139:141-148:150-159:172-178:180-220	1.4	99	92	50	99
GLCE_HUMAN	6I02	B	516	102-617	0.4	100	97	84	100
GLDN_HUMAN	5YBY	A	245	299:302-545	0.4	100	96	44	100
GLIP1_HUMAN	3Q2U	A	193	21:23-214	1	100	97	73	100
GLT10_HUMAN	2D7R	A	536	68-603	0.5	100	96	89	100
GLT12_HUMAN	6PXU	B	532	50-581	0.5	100	97	92	100
GP1BA_HUMAN	1OOK	G	280	17-286:632:635-637:639-642:644-645	2	96	93	43	99
GP1BB_HUMAN	3RFE	A	119	25-143	0.3	99	95	58	100
GPIX_HUMAN	3REZ	A	111	18:20-28:34-103:106-111:113-137	1.3	57	91	63	98
GPVI_HUMAN	5OU9	A	176	20-121:124:127-148:150-159:164-171:175-207	2.2	89	94	52	98
GPX8_HUMAN	3CYN	A	173	37-38:40-210	1.6	96	97	83	99
HAVR1_HUMAN	5DZO	A	107	21-127	0.9	99	93	29	100
HAVR2_HUMAN	6TXZ	A	109	22-130	0.9	100	92	36	100
HEPS_HUMAN	1Z8G	A	364	49-159:167-419	0.6	100	96	87	99
HERP2_HUMAN	2KDB	A	77	9-85	1.2	100	89	19	100
HFE_HUMAN	1A6Z	A	270	26-41:46-299	1.6	100	94	78	99
HLAB_HUMAN	6UJ7	A	279	25-303	1.3	100	97	77	100
HLAC_HUMAN	1EFX	A	278	25-299:301-303	1.9	91	96	76	100

Table 1. Verification of AF2-generated models of 788 bitopic proteins from *Homo sapiens* by comparison with experimental structures from PDB (continuation).

UniProt ID	PDB ID[1]	Chain	N_{over}[2]	Overlapping segments[2]	RMSD, Å [3]	% idenity[4]	Score[5]	%AF2[6]	%PDB[7]
HLAE_HUMAN	3BZF	A	276	20:23-297	1.4	99	96	77	100
HLAF_HUMAN	5KNM	A	276	22-297	0.8	100	97	80	100
HLAG_HUMAN	6AEE	A	275	26-300	0.8	99	97	81	100
HMOX1_HUMAN	1NI6	C	223	1-223	1.1	100	95	77	100
HMOX2_HUMAN	2RGZ	B	219	28:31-247:249	0.8	99	96	69	100
HMR1_HUMAN	6PUM	C	269	22-269:271:273-292	1	99	96	79	100
HS3S5_HUMAN	3BD9	A	258	85-306:311-346	0.7	98	96	75	100
HS3SA_HUMAN	1T8U	B	271	27:31:34:136:140-406	1.4	98	96	67	100
HTRA2_HUMAN	5FHT	A	296	142-279:281:291-346:360-460	1	99	92	65	99
I10R1_HUMAN	1LQS	R	207	21:24-229	1.2	97	94	36	100
I10R2_HUMAN	3LQM	A	199	20-218	1.3	97	95	61	99
I11RA_HUMAN	6O4P	A	295	23-24:26-153:162-323:325-327	1.3	98	93	70	100
I12R1_HUMAN	6WDP	A	207	27-31:33-36:39:41-45:47-58:60-66:68-74:76-123:125-129:131-242:244	3.4	84	84	31	98
I13R1_HUMAN	5E4E	C	303	32-191:198-340	0.8	100	91	71	100
I13R2_HUMAN	3LB6	D	284	29-58:63:65-81:83-98:100-160:164:166-248:253-297:302-331	0.8	100	94	75	100
I15RA_HUMAN	2Z3Q	B	81	28-108	1.8	96	93	30	100
I17RA_HUMAN	4HSA	C	272	33-304	1.4	99	90	31	100
I17RC_HUMAN	6HG4	B	427	80-94:107-169:188-536	1.9	95	83	54	100
I18RA_HUMAN	3WO4	C	270	28-35:37:39-51:77-79:101-102:104-127:138-356	1.2	95	87	45	100
I20RA_HUMAN	4DOH	R	204	39-242	1.1	99	94	37	100
I20RB_HUMAN	4DOH	B	193	34-226	1	99	95	62	100
I22R1_HUMAN	3DGC	S	207	19-225	0.7	99	94	36	100
ICAM1_HUMAN	1Z7Z	I	430	28-334:348-349:353-423:425-433:437-444:448-456:458-481	3	94	94	81	99
ICAM2_HUMAN	1ZXQ	A	192	25-216	1.2	100	94	70	100

Table 1. Verification of AF2-generated models of 788 bitopic proteins from *Homo sapiens* by comparison with experimental structures from PDB (continuation).

UniProt ID	PDB ID[1]	Chain	N_{over}[2]	Overlapping segments[2]	RMSD, Å [3]	% idenity[4]	Score[5]	%AF2[6]	%PDB[7]
ICAM3_HUMAN	1T0P	B	86	30-115	0.9	99	92	16	100
ICAM5_HUMAN	4OI9	A	378	32-406:476-478	1.7	99	92	41	100
ICOS_HUMAN	7JOO	C	102	30-73:77-85:87:90-137	1.9	92	86	51	94
ICOSL_HUMAN	6X4T	A	210	17:19-144:146-150:154-231	1.6	97	93	70	100
IGF1R_HUMAN	5U8Q	A	800	31-64:67:69:71:73-170:172-177:179-184:190-197:199-219:223-674:715-716:718-723:726-734:775-929	3.3	96	88	59	100
IGHD_HUMAN	1ZVO	C	213	390-395:446-476:478-482:484-493:495-497:499-510:513-540:542-553:555-570:572-575:578:580-609:611-620:622-653:655-658:660-668	3.9	66	80	55	42
IGHM_HUMAN	2RCJ	O	229	401:455-463:466-472:475:478:482:485:508-527:529-543:545-554:556-572:574-616:618-636:638-667:670-680:682-683:685-725	3.9	27	90	51	44
IL18R_HUMAN	3WO4	B	301	18-318	1.3	99	89	56	100
IL1AP_HUMAN	3O4O	B	321	24-48:50-57:59-76:78-87:89-339:341-349	1.7	92	93	56	99
IL1R1_HUMAN	1ITB	B	310	23-332	1.2	100	93	54	100
IL1R2_HUMAN	3O4O	C	315	28-299:301-319:321-343:345	1.7	94	90	79	100
IL21R_HUMAN	3TGX	C	209	20-228	0.6	98	93	39	100
IL23R_HUMAN	6WDQ	C	293	23-24:26-316	1.7	99	94	47	100
IL2RA_HUMAN	6VWU	A	136	13-16:139-207:246-308	2.4	94	90	50	53
IL2RB_HUMAN	2ERJ	B	204	32-234:236	0.6	98	93	37	100
IL2RG_HUMAN	2ERJ	C	195	54-248	0.7	100	93	53	100
IL3RA_HUMAN	6NMY	F	270	25:27-294:296	1.2	99	94	71	100
IL3RB_HUMAN	4NKQ	A	410	24-38:41-52:54-90:92-102:104-438	2.7	91	92	46	100
IL4RA_HUMAN	3BPL	B	202	23-224	0.9	96	93	24	100

Table 1. Verification of AF2-generated models of 788 bitopic proteins from *Homo sapiens* by comparison with experimental structures from PDB (continuation).

UniProt ID	PDB ID[1]	Chain	N_{over}[2]	Overlapping segments[2]	RMSD, Å [3]	% idenity[4]	Score[5]	%AF2[6]	%PDB[7]
IL5RA_HUMAN	6H41	A	309	27-335	1.2	99	94	74	100
IL6RA_HUMAN	1N26	A	299	20-318	0.8	100	94	64	100
IL6RB_HUMAN	3L5H	A	585	24-48:50-104:106-111:113-122:124-612	2.6	100	92	64	100
IL7RA_HUMAN	3UP1	A	201	32-232	0.8	100	94	44	100
ILRL1_HUMAN	4KC3	B	200	21-50:53:56-116:118-125:127-210:214-216:261-263:291-293:326-327:354-358	2.7	88	91	36	72
ILRL2_HUMAN	6U6U	R	195	21-27:29-35:37-123:125-131:133-219	1.7	92	83	34	99
INAR1_HUMAN	3SE4	A	283	32-34:36-48:59-82:84-94:96-108:110-114:116-274:277-329:331-332	1.9	93	92	51	99
INAR2_HUMAN	1N6V	A	205	34-38:41-163:165-168:170-238:241:244-245:248	3.1	96	87	40	97
INGR1_HUMAN	1FG9	C	210	29-154:157-161:163-240:242	1.4	98	91	43	100
INGR2_HUMAN	6E3L	E	213	28-240	0.9	100	95	63	100
INLR1_HUMAN	3OG6	B	200	24-223	0.8	100	92	38	100
IRPL1_HUMAN	5WY8	B	323	27-349	0.8	100	93	46	100
ITA1_HUMAN	1PT6	B	195	171-362:367-369	0.9	97	92	17	100
ITA2_HUMAN	1AOX	A	201	168-364:367-370	1.3	98	89	17	100
ITA2B_HUMAN	3FCS	A	913	32-698:700-793:795:806-870:905-989:991	0.6	100	93	88	100
ITA4_HUMAN	3V4V	A	580	34-524:526-538:540-561:563-574:576-588:594-620:673:719	3.1	96	91	56	100
ITA5_HUMAN	7NWL	A	603	42-644	1.5	100	91	57	100
ITA6_HUMAN	7CEC	A	553	24-221:234-240:251-254:294-567:574-579:586-609:612:615-629:631:638:640-661	1.8	99	91	49	100
ITAL_HUMAN	5E6U	A	583	26-331:337:341-614:752-753	0.8	99	92	50	100
ITAM_HUMAN	1NA5	A	195	114:147-335:337-338:344-346	0.9	97	90	17	100

Table 1. Verification of AF2-generated models of 788 bitopic proteins from *Homo sapiens* by comparison with experimental structures from PDB (continuation).

UniProt ID	PDB ID[1]	Chain	N_{over}[2]	Overlapping segments[2]	RMSD, Å [3]	% idenity[4]	Score[5]	%AF2[6]	%PDB[7]
ITAV_HUMAN	4G1E	A	941	31-649:651-868:870:872-876:878:885:897-992	2.4	100	93	90	98
ITAX_HUMAN	4NEH	A	1084	20-1103	0.8	99	90	93	100
ITB1_HUMAN	4WK4	B	435	24-98:102:105-436:438:440-465	1.5	99	92	55	100
ITB2_HUMAN	5ES4	B	674	22:24-696	2.4	100	89	88	100
ITB3_HUMAN	3IJE	B	695	26:28-716:718-721:723	2	99	90	88	100
ITB4_HUMAN	3F7R	A	200	1126-1325	2.4	100	82	11	93
ITB6_HUMAN	4UM9	D	455	22-47:49:56:58-458:460-481:488-491	0.9	99	90	58	100
ITB7_HUMAN	3V4V	B	375	100-474	1.2	100	91	47	100
ITB8_HUMAN	6UJC	B	359	104-439:441:446-467	1.1	100	90	47	100
IZUM1_HUMAN	5JK9	A	240	18-257	1.6	98	93	69	100
JAG1_HUMAN	4CC1	A	306	32-337	1.3	99	88	25	100
JAG2_HUMAN	5MW7	A	289	27-51:62:65-113:116-117:122-156:160-161:163-337	2.8	99	85	23	99
JAM1_HUMAN	1NBQ	A	209	25-233	1.2	100	96	70	100
JTB_HUMAN	2KJX	A	60	48-102:104-106:108-109	2.2	90	85	41	92
K0319_HUMAN	2E7M	A	103	331-333:335-431:433-435	2.3	94	77	10	91
KCNE1_HUMAN	2K21	A	79	3-24:26-28:33-36:38-39:51-52:54-84:89:91-99:102-106	3.6	70	74	61	61
KCNE3_HUMAN	2NDJ	A	54	36-38:41:44-46:48-56:58-67:69-70:75:82:86-87:90-91:94-95:98-102:104-115:118	4.2	7	65	52	52
KI2L1_HUMAN	1NKR	A	195	27-221	1.8	100	95	56	100
KI2L2_HUMAN	2DL2	A	197	25-221	1.6	100	94	57	100
KI2L3_HUMAN	1B6U	A	199	26-223:225	1.1	100	94	58	100
KI2L4_HUMAN	3WYR	A	189	30-218	1.6	99	91	50	100
KI2S2_HUMAN	1M4K	A	191	29-176:181-223	2.2	100	95	63	99
KI2S4_HUMAN	3H8N	A	195	27-221	1.2	100	96	64	100

Table 1. Verification of AF2-generated models of 788 bitopic proteins from *Homo sapiens* by comparison with experimental structures from PDB (continuation).

UniProt ID	PDB ID[1]	Chain	N_{over}[2]	Overlapping segments[2]	RMSD, Å [3]	% idenity[4]	Score[5]	%AF2[6]	%PDB[7]
KI3L1_HUMAN	7K80	G	289	27-66:68-78:80-317	1.6	96	89	65	100
KIRR3_HUMAN	2CRY	A	116	379-383:398:412-521	2.4	94	90	15	95
KIT_HUMAN	2E9W	B	469	32:34-317:320-444:446-447:449-450:452-467:469-506:508	1.9	95	89	48	100
KLOT_HUMAN	5W21	A	914	34-97:116-956:959-967	1.8	99	92	90	99
KLOTB_HUMAN	5VAQ	A	861	52:54-62:75-119:126-537:576-969	0.8	100	96	82	100
KLRB1_HUMAN	5MGS	H	129	90-218	1.6	100	95	57	100
KLRD1_HUMAN	3CDG	J	123	57-179	0.7	100	96	69	100
KLRF2_HUMAN	4IOP	B	121	76-196	0.6	100	96	58	100
KLRG1_HUMAN	3FF7	C	112	75-186	0.4	99	96	57	100
KREM1_HUMAN	5FWV	A	294	29-322	0.7	100	97	62	100
LAIR1_HUMAN	3KGR	A	99	25-123	0.6	99	97	34	100
LAMP3_HUMAN	4AKM	B	157	222-377:379	1.2	99	95	38	100
LCAP_HUMAN	5MJ6	A	860	151:153-154:160-639:649-1025	0.5	100	97	84	99
LDLR_HUMAN	1N7D	A	500	236-239:252-280:282-286:292-295:297-300:395-434:436-547:549-597:599-612:615-853	4.1	82	87	58	78
LEPR_HUMAN	3V6O	B	184	430-451:453-454:460-559:566-588:595-631	0.9	99	88	16	100
LFA3_HUMAN	1CCZ	A	167	28:30-135:138-145:148:150-152:155-164:166-170:175-180:183-186:188-207:210:212-213	2	61	93	67	98
LIFR_HUMAN	3E0G	A	478	51:53-115:119-130:132-180:182-195:197-535	1.8	94	89	44	100
LIGO1_HUMAN	4OQT	A	475	42-516	0.9	100	97	77	100
LIRA2_HUMAN	2OTP	B	142	26-66:69-76:79-160:163:165-172:210-211	2.2	89	92	29	73
LIRA5_HUMAN	2D3V	A	177	43:45-105:107-146:158-176:180-210:212-236	1.2	99	94	59	100

Table 1. Verification of AF2-generated models of 788 bitopic proteins from *Homo sapiens* by comparison with experimental structures from PDB (continuation).

UniProt ID	PDB ID[1]	Chain	N_{over}[2]	Overlapping segments[2]	RMSD, Å [3]	% idenity[4]	Score[5]	%AF2[6]	%PDB[7]
LIRB1_HUMAN	6ZDX	B	396	25-420	1.2	100	92	61	100
LIRB2_HUMAN	6AED	A	385	24:26-63:68-78:81-82:85-162:169-423	2	96	91	64	99
LIRB4_HUMAN	3P2T	A	193	25-163:165-174:176-219	2.5	95	89	43	99
LMAN1_HUMAN	3LCP	A	234	41-274	0.3	100	96	46	100
LMTK3_HUMAN	6SEQ	A	278	128:130:132-227:229:232-235:237-318:321-413	1.1	97	90	19	100
LRBA_HUMAN	1T77	A	414	875:877-1289	0.8	100	94	30	100
LRC32_HUMAN	6GFF	I	551	18:21-280:290-299:312-587:589-592	1	99	91	83	100
LRC4C_HUMAN	3ZYJ	A	393	45-341:343:346-367:369-413:416-423:426-433:435-446	3.2	90	94	61	99
LRFN5_HUMAN	5XNP	A	361	13-14:16-374	1	99	94	50	100
LRIG1_HUMAN	4U7L	A	453	40:42-492:552	1.1	98	94	41	100
LRP1_HUMAN	2FYJ	A	71	258:291-301:304-310:312-316:318-322:324-334:336:338-341:344-348:351-362:365-373	3.9	37	72	5	87
LRP6_HUMAN	4DG6	A	614	20-632:649	1.5	100	90	38	100
LRP8_HUMAN	5B4X	B	441	175:186:188-190:200:203-206:217-226:228-230:235-238:241-243:317:322-324:326:332:337-341:375-380:382-775	2.9	90	90	46	92
LRRC4_HUMAN	3ZYI	A	395	44-319:323-441	0.7	100	96	60	100
LRRT2_HUMAN	5Z8Y	A	328	34-361	0.6	100	96	64	100
LYAM1_HUMAN	3CFW	A	156	39-194	0.8	100	95	42	100
LYAM2_HUMAN	4C16	B	279	21-32:34-273:275-280:282-295:297-302:352	3.5	91	92	46	100
LYAM3_HUMAN	1G1R	D	160	42-198:222:244-245	1.4	98	89	19	100
MA1B1_HUMAN	5KK7	A	455	245-699	0.2	100	98	65	100
MADCA_HUMAN	1BQS	A	206	23-62:64-67:69-164:166-177:179-232	2	94	91	54	99

Table 1. Verification of AF2-generated models of 788 bitopic proteins from *Homo sapiens* by comparison with experimental structures from PDB (continuation).

UniProt ID	PDB ID[1]	Chain	N_{over}[2]	Overlapping segments[2]	RMSD, Å [3]	% idenity[4]	Score[5]	%AF2[6]	%PDB[7]
MANEA_HUMAN	6ZJ5	AAA	360	99-458	0.8	100	97	78	99
MARC1_HUMAN	6FW2	A	285	52-129:289-495	0.4	99	97	85	64
MAVS_HUMAN	2MS8	A	100	3-102	1.7	100	94	19	98
MCL1_HUMAN	6YBL	A	167	338-339:400-407:489-490:492-493:514-515:517-667	2.6	90	88	48	33
MCP_HUMAN	3O8E	B	252	35-286	1.3	100	96	64	100
MEP1B_HUMAN	4GWM	A	561	25-193:200-301:308-596:598	1	100	95	80	100
MERTK_HUMAN	7AAZ	A	286	574-623:625-632:634-751:754-755:758-761:763-764:766-867	2.5	95	88	29	99
MET_HUMAN	7MO7	B	697	25-31:33-104:106-107:110-302:306-307:311-619:621-627:634-659:661-670:672-715:717-720:722-729:731-739:770-771:773:797	3.2	94	88	50	100
MGA_HUMAN	3TOP	A	890	960-1849	0.7	100	95	48	100
MGAT2_HUMAN	5VCM	B	354	84-87:90-298:301-403:410-418:420:422-449	1.6	99	95	79	99
MGT5A_HUMAN	5ZIB	A	572	75-84:131-133:147-151:153:156-174:179-180:182-190:192-213:216-217:219-285:302-336:344-346:352-487:491-748	3.2	89	90	77	99
MICA_HUMAN	1HYR	C	271	23-60:62-63:65-75:77:79-244:246-251:253-276:278-290:292:294-302	3.5	85	93	71	99
MICB_HUMAN	1JE6	A	275	23-297	2.6	98	92	72	100
MICU1_HUMAN	6XJX	Q	361	104-254:256:258-275:277-281:283-337:339:341:344-356:361-475:477	1.7	90	88	76	98
MICU2_HUMAN	6LB7	D	303	76:85-208:210:215:235-409:411	2.2	99	89	70	97
MICU3_HUMAN	6AGI	B	321	135-181:183-270:273-291:336:338-500:502:504-505	1.8	99	89	61	100
MID49_HUMAN	5WP9	B	328	126-283:285-295:297-455	1.8	98	91	72	100
MID51_HUMAN	4NXV	C	333	134-466	1.5	100	94	72	99
MIRO1_HUMAN	5KU1	A	398	181-479:484-582	0.7	100	96	64	100

Table 1. Verification of AF2-generated models of 788 bitopic proteins from *Homo sapiens* by comparison with experimental structures from PDB (continuation).

UniProt ID	PDB ID[1]	Chain	N_{over}[2]	Overlapping segments[2]	RMSD, Å [3]	% idenity[4]	Score[5]	%AF2[6]	%PDB[7]
MIRO2_HUMAN	5KUT	A	172	414-450:455-479:482-487:490-588:591-595	2	94	84	28	96
MMP14_HUMAN	6CM1	A	196	316-511	1.1	100	94	34	100
MMP16_HUMAN	1RM8	A	169	123:125-292	1.2	99	88	28	100
MPEG1_HUMAN	6U2K	B	604	28-435:437:447-544:547:551-646	1.2	100	89	84	100
MPRI_HUMAN	6P8I	A	663	43-117:119:122:124-331:336-346:351-367:369-371:374:377-388:391-491:493-502:504:510-518:520-523:528-532:542-543:548:550:552-554:557-570:572:582-603:607:609-765:819:822	3.5	94	78	27	100
MPZL1_HUMAN	6IGO	C	122	37-156:159-160	0.7	98	92	45	100
MRC1_HUMAN	6INN	A	588	21:23-30:32-41:43-67:69-131:133-156:158-349:352-433:435-466:468-488:490-492:500:502-505:507-522:524-592:594-604:606-619:621-632	3.3	93	87	40	100
MRC2_HUMAN	5EW6	A	442	37-99:103-138:154:156:158-363:365:375:378:380-511	1.6	98	85	30	100
MSPD2_HUMAN	6TQT	A	130	313:315-319:321-341:344-446	1.6	96	85	25	99
MUC1_HUMAN	2ACM	A	57	1041-1097	1.2	100	86	5	100
MUC18_HUMAN	6LYN	C	178	306-311:342-395:397-411:413:415-429:431-446:448-458:460-469:471-480:483-495:497-522:524	3.9	61	91	28	97
MUL1_HUMAN	6M2D	B	56	294:297-351	1.6	98	88	16	98
MXRA8_HUMAN	6JO8	O	258	31-36:38-46:48-79:82-292	3	94	90	58	100
MYOF_HUMAN	2DMH	A	130	8-80:82-85:87-138:349	1.8	95	86	6	93
MYP0_HUMAN	3OAI	A	121	397-516:518	1.1	98	95	49	25
MYRF_HUMAN	5YHU	A	191	342-532	0.3	100	93	17	100
NALD2_HUMAN	3FED	A	690	46-132:136-328:330-739	0.8	100	97	93	100
NALDL_HUMAN	4TWE	A	706	34:36-740	0.7	100	96	95	100

Table 1. Verification of AF2-generated models of 788 bitopic proteins from *Homo sapiens* by comparison with experimental structures from PDB (continuation).

UniProt ID	PDB ID[1]	Chain	N_{over}[2]	Overlapping segments[2]	RMSD, Å [3]	% idenity[4]	Score[5]	%AF2[6]	%PDB[7]
NB5R3_HUMAN	1UMK	A	271	31-301	0.7	100	98	90	100
NCAM1_HUMAN	3MTR	A	197	411-607	0.8	98	94	23	100
NCAM2_HUMAN	2JLL	A	376	300:302-345:347:349-462:464-467:469-620:622-634:638-639:641-647:650:652-656:658-662:664-666:668-691	3.6	85	94	45	99
NCLN_HUMAN	6W6L	5	465	45-59:61-72:84-119:126:130-135:145-181:193-194:197-209:221-224:226:228-325:329-334:336-337:340-362:364:367-368:370-384:386-399:405-465:468:471-476:479-483:486-501:503-505:518-536:539-548:554-606:608-609:612	4.8	72	90	83	90
NCPR_HUMAN	5EMN	A	607	67-237:242-677	0.7	100	95	90	100
NCTR1_HUMAN	1P6F	A	188	25-212	0.3	100	97	62	100
NCTR2_HUMAN	1HKF	A	108	23-130	0.9	100	96	39	100
NCTR3_HUMAN	3NOI	A	115	20-134	1.2	98	95	57	97
NDST1_HUMAN	1NST	A	282	579-586:601-664:670-879	0.6	100	96	32	100
NDUA1_HUMAN	5XTI	S	70	1-70	0.9	100	97	100	100
NDUA3_HUMAN	5XTI	U	83	2-84	0.9	100	97	99	100
NDUA4_HUMAN	5Z62	N	77	1:4-44:46-51:53-75:77-82	2	58	93	95	97
NDUAD_HUMAN	5XTI	W	132	13-16:20-21:23-33:35-38:40-150	1.8	92	96	92	96
NDUB1_HUMAN	5XTI	n	55	3-51:53-55:57-59	1.6	96	93	95	98
NDUB3_HUMAN	5XTI	Z	79	10-51:53-57:59-89:91	0.9	94	93	81	99
NDUB4_HUMAN	5XTI	o	128	2-129	1.4	100	95	99	100
NDUB5_HUMAN	5XTI	a	138	52-189	1.1	100	95	73	100
NDUB6_HUMAN	5XTI	b	101	40:45:49-110:112-114:116-130:132-134:136-151	2.7	76	89	79	81
NDUB8_HUMAN	5XTI	c	152	33:35-178:180-184:186-187	1.2	97	95	82	99
NDUBB_HUMAN	5XTI	e	97	53-54:56-149:151	1.9	97	90	63	100

Table 1. Verification of AF2-generated models of 788 bitopic proteins from *Homo sapiens* by comparison with experimental structures from PDB (continuation).

UniProt ID	PDB ID[1]	Chain	N_{over}[2]	Overlapping segments[2]	RMSD, Å [3]	% idenity[4]	Score[5]	%AF2[6]	%PDB[7]
NDUC2_HUMAN	5XTI	g	118	1:3-5:7-120	1.4	98	91	99	99
NECT1_HUMAN	4MYW	B	303	33-334:336	2	100	94	59	100
NECT2_HUMAN	5V52	D	127	33-108:110-116:119-160:162:187	1.5	92	89	24	100
NECT3_HUMAN	4FOM	A	302	58-357:359-360	1.4	100	94	55	100
NECT4_HUMAN	4FRW	A	215	32-244:321-322	1.4	99	93	42	100
NEO1_HUMAN	3P4L	A	198	852-898:900:903-1052	1.7	99	81	14	100
NEP_HUMAN	6SUK	A	698	53-750	0.2	100	98	93	100
NFASC_HUMAN	3P40	A	390	37-426	1.4	100	88	29	100
NICA_HUMAN	7D8X	A	667	34-699:701	1.6	100	91	94	100
NKG2A_HUMAN	3CDG	F	120	110-111:115-232	1	98	93	52	100
NKG2D_HUMAN	1MPU	A	128	85-86:88-89:91:93-215	1	97	94	59	100
NLGN1_HUMAN	5OJ6	A	547	50:52-164:205:207-208:211-317:327-599:607-656	0.7	99	95	63	100
NLGN2_HUMAN	5XEQ	A	525	42-149:175-392:401-552:563-609	0.8	100	95	63	100
NLGNX_HUMAN	2WQZ	A	545	35-36:38-160:164-539:543:555:557-596:598-599	1.1	98	94	67	100
NOTC1_HUMAN	6PY8	F	249	1555-1556:1566:1569-1573:1732:1735:1741:1744-1745:1748:1878:1880-1896:1906:1909-2124	2.3	94	80	10	98
NOTC2_HUMAN	2OO4	A	226	1424-1426:1428-1594:1617-1672	1.3	98	77	9	100
NOTC3_HUMAN	4ZLP	A	241	1385-1562:1575-1637	1.7	100	79	10	100
NPTN_HUMAN	6A69	B	127	236-361:364	1.3	100	95	32	100
NR3L1_HUMAN	6YJP	C	224	22:24-151:153-157:159-248	1.2	96	92	49	100
NRCAM_HUMAN	1UEN	A	123	574-577:619-620:623-624:759-760:842-895:898-952:954-956:977	2.4	90	86	9	98
NRG1_HUMAN	1HRE	A	57	172-174:177:179-200:202-205:207-218:220:222-229:231-233:235-236:628	3.1	79	76	9	85

Table 1. Verification of AF2-generated models of 788 bitopic proteins from *Homo sapiens* by comparison with experimental structures from PDB (continuation).

UniProt ID	PDB ID[1]	Chain	N_{over}[2]	Overlapping segments[2]	RMSD, Å [3]	% idenity[4]	Score[5]	%AF2[6]	%PDB[7]
NRP1_HUMAN	2QQM	A	436	145-197:200-201:204-270:273-586	1	99	92	47	100
NRP2_HUMAN	2QQL	A	554	26-196:208-508:516:518-595:597:600:602	1.2	99	93	60	100
NSDHL_HUMAN	6JKG	A	193	35-150:165:167:169-203:211:228:234-237:261-291:293:374:377	2.1	94	96	52	89
NTRK1_HUMAN	2IFG	B	343	35-44:46-61:63-67:69-81:83-92:94-233:235-242:244-320:322-326:328-383:385:391-392	4.2	88	86	43	99
NTRK2_HUMAN	4AT5	A	288	524-560:566-816	2.2	99	86	35	98
NTRK3_HUMAN	6KZD	A	276	528-636:649-712:729-831	1.3	100	87	33	98
OLR1_HUMAN	6TL7	B	133	142-274	1	98	94	49	99
OSBL8_HUMAN	1V88	A	126	150-274:277	2.4	94	82	14	97
OST48_HUMAN	6S7O	G	411	42-452	0.7	100	95	90	100
OSTM1_HUMAN	7JM7	B	195	81-135:137:146-208:210:221-282:286-287:289-297:300-301	2.4	92	90	58	89
OTULL_HUMAN	6DRM	A	270	87-356	1.1	100	95	76	100
P4HTM_HUMAN	6TP5	A	370	107-180:182:187-481	0.6	100	92	74	100
PAR16_HUMAN	6HXS	A	248	3-225:227-228:252-274	1.6	98	92	77	100
PCD15_HUMAN	6E8F	C	249	9-13:295-301:316-322:359-364:377-381:383-386:415-419:454-461:463-464:466-471:473-510:518-595:606-650:653-658:661-687	4.4	76	85	13	75
PCD17_HUMAN	6VFT	C	420	17:19-41:44-46:60-393:408-464:500:504	2.2	98	90	36	100
PCD18_HUMAN	6VFR	A	428	27:29-455	2.5	100	89	38	100
PCD19_HUMAN	6VFU	C	427	22-49:51-57:59-448:483-484	2.7	97	91	37	100
PCDGF_HUMAN	6MEQ	A	416	30:32-445:539	2	99	91	45	100
PCDH1_HUMAN	6VFP	A	439	59-497	2.6	100	88	41	100

Table 1. Verification of AF2-generated models of 788 bitopic proteins from *Homo sapiens* by comparison with experimental structures from PDB (continuation).

UniProt ID	PDB ID[1]	Chain	N_{over}[2]	Overlapping segments[2]	RMSD, Å [3]	% idenity[4]	Score[5]	%AF2[6]	%PDB[7]
PCDH7_HUMAN	2YST	A	108	276:310-341:343:347-348:351-399:401-403:405-420:466:522-524	2.4	92	92	10	91
PCDH8_HUMAN	6VFV	A	222	489-491:493-711	1.8	99	90	21	100
PCDH9_HUMAN	2EE0	A	113	218-219:240-245:247-279:281-285:287-353	1.9	89	90	9	99
PD1L1_HUMAN	3FN3	A	215	17:19-232	1.4	100	95	74	100
PD1L2_HUMAN	6UMT	B	99	20-63:65:68-121	1	99	93	36	100
PDCD1_HUMAN	6JJP	F	118	29-146	1	100	92	41	100
PECA1_HUMAN	5GNI	B	198	31-65:67-212:214-224:226-231	1.2	90	90	27	100
PEX14_HUMAN	2W84	A	57	17:19-20:22:24-73:75-77	2	88	85	15	100
PGAM5_HUMAN	5MUF	A	210	56-63:65-67:91-289	0.5	99	97	73	100
PGFRA_HUMAN	5K5X	A	343	554-697:776-785:787-975	1.1	97	84	32	99
PGFRB_HUMAN	3MJG	X	274	32-36:38-104:110-111:113-312	1.6	98	86	25	100
PGRC1_HUMAN	4X8Y	A	111	70-180	0.9	97	96	57	99
PIGR_HUMAN	6UE9	C	536	20-507:509-514:525-565:567	1.9	99	89	70	100
PILRA_HUMAN	5XOF	B	120	31-150	0.5	98	95	40	100
PILRB_HUMAN	4NFD	A	120	31-150	0.5	98	97	53	100
PLA2R_HUMAN	6JLI	A	118	1117-1234	0.8	100	81	8	100
PLAT2_HUMAN	4DPZ	X	108	2-39:52-105:111-112:114-127	1.6	100	90	67	100
PLAT3_HUMAN	2KYT	A	122	5-42:44-54:56-128	2.3	92	87	75	98
PLAT4_HUMAN	2LKT	A	117	1-39:52-53:56-59:61-63:65-133	3.1	94	85	71	94
PLM_HUMAN	2JO1	A	59	31-32:37-73:79-80:83:86-88:90-91:93-103:105	3.4	70	72	64	82
PLXA2_HUMAN	3Q3J	A	100	1288:1291:1494-1495:1498-1540:1546-1576:1581-1602	1.5	96	85	5	98
PLXA4_HUMAN	4.00E+74	A	101	1496-1507:1512-1574:1578-1602:1604	1	99	85	5	100

Table 1. Verification of AF2-generated models of 788 bitopic proteins from *Homo sapiens* by comparison with experimental structures from PDB (continuation).

UniProt ID	PDB ID[1]	Chain	N_{over}[2]	Overlapping segments[2]	RMSD, Å [3]	% idenity[4]	Score[5]	%AF2[6]	%PDB[7]
PLXB1_HUMAN	3SU8	X	524	1558-1559:1562-1757:1764-1854:1879:1881-1896:1898-1899:1929-2144	1.8	99	84	25	97
PLXB2_HUMAN	4.00E+71	A	90	1459-1500:1510-1519:1521-1535:1539-1561	1	100	86	5	100
PLXC1_HUMAN	6VXK	B	737	37-87:89-93:96:112-520:529-544:546:548:551:553:556-559:564-565:567:582-595:668-797:799:802-822:829-845:847-859:871-891:903-917:934-945	3	93	82	47	99
PLXD1_HUMAN	3H6N	A	101	1553-1564:1566-1574:1576-1651:1653-1656	1.5	98	86	5	100
PMGT1_HUMAN	5GGI	B	551	97-647	0.5	100	97	83	100
PMGT2_HUMAN	6XFI	A	511	52-278:285-465:477:479-580	1.5	100	95	88	100
PPAP_HUMAN	1ND6	B	343	33-374:376	0.3	100	98	89	100
PREB_HUMAN	5TF2	A	337	8-35:37-40:42-55:57-99:138-236:239-387	1.3	99	95	81	100
PRLR_HUMAN	3NCF	B	206	25-230	0.4	99	94	33	100
PRSS8_HUMAN	3E0N	B	249	49-297	1.2	99	94	73	99
PTCRA_HUMAN	3OF6	E	104	23-126	0.9	100	88	37	100
PTGIS_HUMAN	2IAG	A	470	23-319:328-500	0.7	100	96	94	100
PTK7_HUMAN	6VG3	B	279	786:789-1066	1.8	100	85	26	100
PTN1_HUMAN	7L0C	A	299	1-299	0.3	100	97	69	100
PTN2_HUMAN	1L8K	A	273	5-276:278	1.3	100	96	66	100
PTPR2_HUMAN	2QEP	A	288	723:725-968:970-1012	1	99	93	28	100
PTPRA_HUMAN	6UZT	A	587	215-801	1.3	100	96	73	100
PTPRB_HUMAN	2I4G	A	288	1678-1749:1754-1853:1856-1971	1.3	100	89	14	100
PTPRC_HUMAN	1YGU	B	567	623:626-627:629-738:740-749:751-768:770-868:870-894:896-931:933-989:993:1015-1023:1025-1031:1033-1048:1050-1059:1061-1139:1141-1153:1156-1159:1162-1212:1214-1232	1.4	98	92	43	100

Table 1. Verification of AF2-generated models of 788 bitopic proteins from *Homo sapiens* by comparison with experimental structures from PDB (continuation).

UniProt ID	PDB ID[1]	Chain	N_{over}[2]	Overlapping segments[2]	RMSD, Å [3]	% idenity[4]	Score[5]	%AF2[6]	%PDB[7]
PTPRD_HUMAN	4RCA	A	297	21-232:234-248:250-272:275-282:285-308:311-324:402	3.2	88	85	16	99
PTPRE_HUMAN	2JJD	F	542	111-129:132-181:183-281:288-406:425-472:479-574:581-691	1.5	100	95	77	100
PTPRF_HUMAN	6KR4	A	575	1328-1650:1654-1903:1905-1906	0.8	99	91	30	100
PTPRG_HUMAN	2NLK	A	553	827-897:903-1142:1148-1160:1167-1173:1175-1196:1204-1250:1260-1412	0.6	99	94	38	100
PTPRJ_HUMAN	2NZ6	A	279	1024:1026-1027:1030-1155:1166-1241:1247-1318:1322:1325	1.5	96	93	21	94
PTPRK_HUMAN	2C7S	A	289	864-932:935-1147:1176-1177:1180-1184	1	97	92	20	100
PTPRM_HUMAN	2V5Y	A	564	22-525:528:530-582:614-618:671	3.4	99	91	39	100
PTPRN_HUMAN	2I1Y	B	290	687-976	0.5	100	94	30	100
PTPRO_HUMAN	2GJT	A	295	914-1208	0.8	99	93	24	100
PTPRQ_HUMAN	4IKC	A	274	2013-2137:2145-2293	1.1	99	85	12	100
PTPRR_HUMAN	2A8B	A	283	373-655	1.1	99	95	43	100
PTPRS_HUMAN	2FH7	A	568	1367-1688:1697-1942	1	100	91	29	100
PTPRT_HUMAN	2OOQ	B	281	866-1146	1	99	93	20	100
PTPRU_HUMAN	6SUB	A	253	872-903:926-1146	1.3	100	91	18	100
PTPRZ_HUMAN	5AWX	A	283	1696-1727:1731-1732:1734:1738-1877:1888-1995	0.9	96	91	12	100
PVR_HUMAN	3J8F	7	306	28-330:332-333:335	1.2	99	94	73	100
PYRD_HUMAN	6IDJ	A	367	28-29:31-395	1.1	100	97	93	100
QCR10_HUMAN	5XTI	AG	51	2-48:50-53	0.7	92	92	91	100
QCR8_HUMAN	5XTI	AA	78	2-71:73-74:76:78-82	1.2	90	95	95	96
QCR9_HUMAN	5XTI	AD	62	2-63	1.3	100	95	98	100
QPCTL_HUMAN	3PB8	X	313	69:71-382	0.5	100	97	82	100
QSOX1_HUMAN	3LLI	A	250	294-517:520-545	1.5	100	95	33	100

Table 1. Verification of AF2-generated models of 788 bitopic proteins from *Homo sapiens* by comparison with experimental structures from PDB (continuation).

UniProt ID	PDB ID[1]	Chain	N_{over}[2]	Overlapping segments[2]	RMSD, Å [3]	% idenity[4]	Score[5]	%AF2[6]	%PDB[7]
R144A_HUMAN	1WIM	A	94	13-102:104-107	1.9	86	90	32	100
RAGE_HUMAN	4LP5	A	301	20-273:275-293:295-322	2.5	93	92	74	100
RAMP1_HUMAN	7KNU	E	115	29-142:144	1	99	96	78	100
RAMP2_HUMAN	6UUN	E	107	60-129:131-134:136-139:141-144:147-171	2.4	69	95	61	99
RAMP3_HUMAN	6UVA	E	111	31:34-137:139-141:143-144:147	2.5	94	95	75	100
RET_HUMAN	6Q2R	E	572	29-128:137-207:211-246:251-379:387-622	0.9	100	91	51	100
RMD3_HUMAN	7CC7	A	227	239-334:337-391:393-468	0.9	100	93	48	100
RN128_HUMAN	3ICU	A	169	38-203:205:207:210	1.5	97	92	39	99
RNF13_HUMAN	5ZC4	A	73	215-287	0.9	99	88	19	100
RNF24_HUMAN	2EP4	A	55	87-137:139-140:142-143	1.7	93	79	37	74
RNF43_HUMAN	4KNG	E	147	44-177:180-192	1.3	91	88	19	99
ROBO1_HUMAN	5OPE	A	382	60:62:65-68:70-115:117-124:126:128-137:139-449	3.3	94	90	23	99
ROBO2_HUMAN	6IAA	A	804	22-82:84-188:190-199:201-207:209-283:285-291:293-301:303-593:595-608:610-670:672-721:723-741:743-750:753-756:758-840	2.1	98	90	58	100
ROBO3_HUMAN	6POK	A	195	668-709:713-833:835-840:842-867	2.8	97	84	14	99
RON_HUMAN	4QT8	B	629	28-357:361-580:583-597:600-603:606-612:614-618:620-621:634-646:648:652-682:684	1.2	96	89	45	100
ROR1_HUMAN	6TU9	A	267	460-568:588-647:652-749	1.3	100	89	28	100
ROR2_HUMAN	4GT4	A	274	464-493:501-511:513-515:517-577:582:584-751	1.4	99	88	29	100
ROS1_HUMAN	4UXL	A	288	1932-1933:1936-2111:2120-2229	0.8	100	84	12	100
RPN1_HUMAN	6S7O	E	552	29-90:92:96-106:109-462:465-467:469-498:501-507:515-516:518-520:522-543:545-574:578-579:585:588-590:594-614	3.9	77	93	91	99

Table 1. Verification of AF2-generated models of 788 bitopic proteins from *Homo sapiens* by comparison with experimental structures from PDB (continuation).

UniProt ID	PDB ID[1]	Chain	N_{over}[2]	Overlapping segments[2]	RMSD, Å [3]	% idenity[4]	Score[5]	%AF2[6]	%PDB[7]
RYK_HUMAN	6TUA	A	294	298-302:310-351:358-498:502-607	0.7	100	94	48	100
SARAF_HUMAN	6O2V	A	134	30-88:91-147:150:324-340	2.1	87	94	40	99
SC22B_HUMAN	3EGD	C	137	1-23:29-128:131-132:134-135:148-157	1	96	89	64	100
SC61G_HUMAN	6W6L	2	62	25020	0.9	100	94	91	100
SCAR5_HUMAN	7C00	A	107	390-496	1.5	97	95	22	99
SCF_HUMAN	1EXZ	B	139	27-154:157-160:162-168	2.6	94	84	51	99
SCN1B_HUMAN	6J8J	B	173	20-191:194	1.5	99	93	79	100
SCN2B_HUMAN	6VRR	A	127	27-153	1.5	96	95	59	100
SCN3B_HUMAN	4L1D	A	113	24:26-51:56-126:134-148	0.9	96	92	53	100
SCN4B_HUMAN	4MZ3	A	117	40-59:61-157	2.5	99	95	51	98
SCO1_HUMAN	2GT6	A	167	139-304:307	1.6	100	94	55	97
SCO2_HUMAN	2RLI	A	163	105-264:266-268	2.7	98	95	61	96
SDK2_HUMAN	1WFO	A	116	1253-1256:1258:1284-1285:1309-1411:1443-1446:1449:1470	2.2	90	84	5	89
SE6L1_HUMAN	2YRA	A	74	662-664:741-805:850-851:855:857-859	1.5	80	83	7	100
SEM4D_HUMAN	1OLZ	A	621	24-220:223:226-648	0.8	100	95	72	100
SEM6A_HUMAN	6WTS	A	536	19-44:54-454:459-563:565-568	0.9	99	95	52	100
SEM7A_HUMAN	3NVQ	A	588	46-632:634	0.6	100	95	88	100
SEPR_HUMAN	6Y0F	C	722	36-757	0.9	100	97	95	100
SGMR1_HUMAN	6DK1	C	205	19:26:32-33:36-236	2.1	100	95	92	93
SGPL1_HUMAN	4Q6R	B	443	110-352:354-553	0.4	100	97	78	100
SHPS1_HUMAN	2WNG	A	307	31-94:98-127:129-318:326-348	1	96	95	61	100
SIA7B_HUMAN	6APL	C	301	65-66:68-140:144-203:205-238:240-297:299-308:310-341:343-374	1.1	98	95	80	100
SIA8C_HUMAN	5CXY	A	292	89-380	0.9	100	96	77	100

Table 1. Verification of AF2-generated models of 788 bitopic proteins from *Homo sapiens* by comparison with experimental structures from PDB (continuation).

UniProt ID	PDB ID[1]	Chain	N_{over}[2]	Overlapping segments[2]	RMSD, Å [3]	% idenity[4]	Score[5]	%AF2[6]	%PDB[7]
SIAT1_HUMAN	4JS2	A	318	89-406	0.3	100	98	78	100
SIGL5_HUMAN	2ZG2	A	214	19-218:220:222-234	1.7	99	89	39	100
SIGL7_HUMAN	1O7V	A	123	24-75:78-148	1.3	100	90	26	97
SIGL8_HUMAN	2N7B	A	145	17-154:156:225-227:234-236	2.1	94	89	29	100
SIRB1_HUMAN	2D9C	A	124	35-151:238-240:242-245	2	93	94	31	91
SIRPG_HUMAN	4I2X	E	314	30-322:325-345	1.6	100	94	81	100
SLAF5_HUMAN	2PKD	E	109	23-131	1.4	100	93	32	100
SLAF6_HUMAN	2IF7	D	192	23-24:26-215	1.7	100	91	58	100
SLC31_HUMAN	6LI9	A	621	61-65:68:71-81:84-687	2.2	98	93	91	100
SLIK1_HUMAN	4RCA	B	241	22-262	0.8	100	91	35	100
SLMAP_HUMAN	6AR0	A	138	2-139	0.9	100	85	17	100
SORC2_HUMAN	1WGO	A	104	362-363:793-893:964	2.2	93	89	9	85
SORL_HUMAN	3WSY	A	653	86-690:699-704:714-722:725:729-735:737-753:755:2152-2153:2156-2158:2161:2220	2.9	94	85	29	99
SORT_HUMAN	5MRI	A	662	87-748	1.1	100	94	80	100
SPAST_HUMAN	6PEN	D	293	318-610	2	100	92	48	100
SPIT1_HUMAN	5H7V	A	319	46:48-147:149-153:155-157:165:168-225:233-307:324:331:336-337:339-340:351:354:366-367:369-370:372-374:377:379:381:383-385:387-389:391-442	1.8	89	88	60	100
SPIT2_HUMAN	4U32	X	54	130-183	0.5	46	95	21	100
SPTC1_HUMAN	7K0P	E	435	60-61:64-65:67-72:74-75:77-86:89-90:92-502	2.1	95	96	92	94
SPTC2_HUMAN	7CQI	T	495	49-52:55-431:435:437-549	2	99	93	88	99
SRPRB_HUMAN	7NFX	v	186	65-207:229-271	1.1	100	94	69	100
ST14_HUMAN	5LYO	B	251	601-613:615:618-795:797-802:804-856	2.1	96	87	29	100
STAB2_HUMAN	5N86	A	139	2311-2449	1.4	99	76	5	100

Table 1. Verification of AF2-generated models of 788 bitopic proteins from *Homo sapiens* by comparison with experimental structures from PDB (continuation).

UniProt ID	PDB ID[1]	Chain	N_{over}[2]	Overlapping segments[2]	RMSD, Å [3]	% idenity[4]	Score[5]	%AF2[6]	%PDB[7]
STIM1_HUMAN	2K60	A	145	35-37:43-45:62:65-175:177-179:181-184:186-188:190-206	2.7	91	84	21	97
STIM2_HUMAN	2L5Y	A	147	50:53:56:65-208	2.9	98	85	20	98
STX10_HUMAN	4DND	A	92	5-31:41-94:96-101:103-105:107:112	1	99	93	37	100
STX12_HUMAN	2DNX	A	119	25-140:143-144:147	1.8	98	91	43	92
STX17_HUMAN	7BV6	F	50	20:91-92:94-136:138-141	2.5	6	74	17	82
STX6_HUMAN	4J2C	A	108	1-108	1.4	98	87	42	100
SUIS_HUMAN	3LPP	A	871	61-931	0.3	100	96	48	100
SUN1_HUMAN	6R2I	A	179	139-143:148:605-607:642-675:696-831	2.6	96	86	22	90
SUN2_HUMAN	6WMD	A	196	521:523-717	1.5	100	87	27	100
SYCY1_HUMAN	5HA6	B	49	451-452:454-456:458-501	2.7	4	71	9	62
SYCY2_HUMAN	6RX3	C	98	354-360:375:379-468	2.8	92	60	18	99
SYJ2B_HUMAN	2ENO	A	108	10-117	1.7	98	87	74	90
SYNE1_HUMAN	6XF2	A	117	944-945:947-949:951-985:987-988:991-998:1000-1030:1039-1040:1042-1074:1076	3.2	3	92	8	99
SYNE2_HUMAN	6XF1	A	223	172-174:177:234:236-306:308-309:312:314-369:371-372:375-383:386-422:426:428-466	3.5	12	88	16	97
SYT1_HUMAN	2R83	A	167	102-103:155-157:242-245:248-249:334-336:379-531	3.1	92	92	40	60
SYT13_HUMAN	1WFM	A	128	164-255:258-260:262:264-291:294:297:299-300	1.7	94	85	30	93
SYT4_HUMAN	1UGK	A	134	151-284	1.7	93	89	32	97
SYT5_HUMAN	5H4Z	A	129	105-233	1	98	91	33	100
SYT7_HUMAN	2D8K	A	135	137-266:271-274:298	1.6	96	92	34	96
TACT_HUMAN	6ARQ	A	109	27-93:96-136:138	1.3	98	82	19	100
TEN2_HUMAN	6VHH	A	753	624-876:878:881:886-992:1001-1057:1067-1400	1.7	100	94	54	44

Table 1. Verification of AF2-generated models of 788 bitopic proteins from *Homo sapiens* by comparison with experimental structures from PDB (continuation).

UniProt ID	PDB ID[1]	Chain	N_{over}[2]	Overlapping segments[2]	RMSD, Å [3]	% idenity[4]	Score[5]	%AF2[6]	%PDB[7]
TF_HUMAN	6R2W	T	209	35-243	0.8	100	95	71	100
TFR1_HUMAN	6D05	A	641	120-760	1.4	100	94	84	100
TGBR3_HUMAN	7LBG	D	156	47-71:81-211	0.7	100	90	18	100
TGFA_HUMAN	1MOX	C	49	41-88:90	2	98	75	31	100
TGFR1_HUMAN	3KCF	E	330	169:171-173:175-500	1.5	99	92	66	100
TGFR2_HUMAN	7DV6	A	304	240-542:544	0.8	98	94	54	100
TIE1_HUMAN	5N06	A	79	643-685:689-724	1.2	100	86	7	85
TIE2_HUMAN	4K0V	A	514	23-486:488-491:493-537:539	1.1	91	91	46	100
TIGIT_HUMAN	3Q0H	A	109	21-128:130	1.1	99	96	45	100
TIMD4_HUMAN	5DZN	A	114	20:22-134	1.8	99	94	30	100
TLR1_HUMAN	6NIH	B	518	26-484:487-506:529:531-545:547-551:555-561:565-566:568-570:572-574:576:579-580	2.2	89	93	66	100
TLR10_HUMAN	2J67	A	141	627:629-658:660-715:717:724-776	1.1	97	79	17	100
TLR2_HUMAN	6NIG	C	548	27-540:542-558:562-567:572-577:579-581:586-587	1.9	90	93	70	100
TLR3_HUMAN	7C76	A	691	29-32:34-336:343-725:727	1.6	99	96	76	100
TLR4_HUMAN	4G8A	B	605	23-627	1.3	100	95	72	100
TLR5_HUMAN	3J0A	A	594	12-13:16:19-24:32-87:89-195:197:199-210:213-221:223-270:279-443:445-456:458-497:500:503-507:509-524:527:530-531:533-635:637-643	4.8	72	95	69	75
TLR6_HUMAN	4OM7	A	143	640-782	1	100	82	18	100
TLR7_HUMAN	7CYN	A	786	30-31:33-435:471-476:490-856:860-861:863-868	1.2	98	94	75	100
TLR8_HUMAN	4QBZ	A	754	31-100:113-434:457-818	1.1	100	91	72	100
TM11E_HUMAN	2OQ5	A	231	194-424	0.8	100	93	55	100
TMED2_HUMAN	5AZW	A	94	20-113	1.2	100	90	47	100

Table 1. Verification of AF2-generated models of 788 bitopic proteins from *Homo sapiens* by comparison with experimental structures from PDB (continuation).

UniProt ID	PDB ID[1]	Chain	N_{over}[2]	Overlapping segments[2]	RMSD, Å [3]	% idenity[4]	Score[5]	%AF2[6]	%PDB[7]
TMPS2_HUMAN	7MEQ	A	325	148-162:166-202:208-215:217:221-247:249-250:258-492	1.2	99	91	66	100
TMX1_HUMAN	1X5E	A	120	31-143:146-152	1.7	91	94	43	95
TMX2_HUMAN	2DJ0	A	129	140-268	1.3	96	94	44	94
TN13B_HUMAN	4ZCH	A	164	80-81:95-98:108-118:147-150:247:394-472:481-543	3.5	87	93	58	39
TNF10_HUMAN	1D4V	B	162	119-135:137-142:144-282	1.4	97	90	58	99
TNF11_HUMAN	5BNQ	A	156	161:163-317	0.4	99	96	49	100
TNF12_HUMAN	4HT1	T	131	105-114:121-194:202:204-249	0.6	99	95	53	100
TNF14_HUMAN	4RSU	G	150	91-240	1.7	99	94	62	100
TNF15_HUMAN	2RE9	A	164	88-251	1.4	100	92	65	100
TNF18_HUMAN	3B93	A	123	77-199	1.3	100	94	62	100
TNFA_HUMAN	7KPB	A	153	81-233	1.4	100	95	66	100
TNFC_HUMAN	4MXW	D	135	87-147:155-165:169-171:173-187:199-243	1.2	90	93	55	100
TNFL4_HUMAN	2HEV	F	126	58-183	0.7	98	93	69	100
TNFL6_HUMAN	5L19	A	145	141-146:148-286	1.3	94	95	52	97
TNFL9_HUMAN	6MGE	B	153	90-111:113:115-118:121-246	1.8	97	92	60	99
TNR14_HUMAN	4RSU	D	105	38-141:143	1.7	98	96	37	100
TNR16_HUMAN	2N80	A	94	333:335-427	2.1	100	82	22	100
TNR17_HUMAN	2KN1	A	48	3-7:9-47:49-51:54	2.5	81	83	26	98
TNR18_HUMAN	7KHD	C	98	39-51:71-155	1.2	100	92	41	100
TNR1A_HUMAN	1EXT	A	159	42-183:185-201	2.7	90	93	35	99
TNR1B_HUMAN	3ALQ	S	164	37-200	0.8	100	97	36	100
TNR21_HUMAN	3U3V	A	164	51-214	0.9	100	96	25	100
TNR3_HUMAN	4MXW	R	149	42:44-106:108:111-112:114-126:128:130-132:142-154:156:158:160-161:163:165-211	2.1	93	91	34	100
TNR4_HUMAN	6OGX	G	140	31-170	1	100	97	51	100

Table 1. Verification of AF2-generated models of 788 bitopic proteins from *Homo sapiens* by comparison with experimental structures from PDB (continuation).

UniProt ID	PDB ID[1]	Chain	N_{over}[2]	Overlapping segments[2]	RMSD, Å [3]	% idenity[4]	Score[5]	%AF2[6]	%PDB[7]
TNR5_HUMAN	5IHL	H	130	22:24-125:132-151:166-167:169-171:190-191	3.8	96	95	47	78
TNR6_HUMAN	1DDF	A	112	223-325:327:330-331:337-342	3.2	92	84	33	88
TNR9_HUMAN	6MGP	X	144	23:25:27-167:169	1.9	95	92	56	100
TOIP1_HUMAN	4TVS	A	224	72:361-583	1.3	100	93	38	100
TOIP2_HUMAN	5J1T	B	234	232:236:238-429:432-471	1.3	99	94	50	100
TOM22_HUMAN	7CP9	G	72	42-45:48:55:58-64:67:72-73:75-107:109-129:131-132	2.8	57	86	51	84
TOM7_HUMAN	7CP9	E	51	4-54	2	100	94	93	98
TOM70_HUMAN	7DHG	C	470	108:110-274:297-599:601	2.2	100	93	77	100
TPBG_HUMAN	4CNM	A	280	63-167:169:172:175-347	1.4	99	93	67	99
TPSN_HUMAN	6ENY	C	364	21-33:37-46:48-49:54-57:59-60:62-187:189:192:195:198-400:402	2.6	97	92	81	100
TPSNR_HUMAN	5WER	C	317	29:31-43:45-46:56-70:72:85-86:88:91-109:111-112:114-115:117-128:138-256:258:262-314:317-339:344-373:379-389:392-401	1.6	86	89	68	100
TPST1_HUMAN	5WRJ	A	275	64-338	0.3	100	98	74	100
TPST2_HUMAN	3AP2	A	300	51-56:60-353	0.3	100	97	80	100
TR10A_HUMAN	5CIR	E	102	128:130-230	1	97	92	22	100
TR10B_HUMAN	1D4V	A	114	75-188	1.6	99	92	26	97
TRAC_HUMAN	6JXR	m	132	108-109:111-152:154-162:164-213:222-223:226-227:229-252:255	3.9	72	92	94	53
TRBC1_HUMAN	4ZDH	B	129	113-241	0.5	95	96	73	54
TRBC2_HUMAN	3T0E	D	130	116-245	0.7	98	96	73	53
TRBM_HUMAN	1DX5	J	116	363-468:471-477:480-482	2.1	94	84	20	98
TRDC_HUMAN	1HXM	A	86	121-206	0.7	100	95	56	42
TREM1_HUMAN	1Q8M	B	119	20-138	2.3	98	89	51	98

Table 1. Verification of AF2-generated models of 788 bitopic proteins from *Homo sapiens* by comparison with experimental structures from PDB (continuation).

UniProt ID	PDB ID[1]	Chain	N_{over}[2]	Overlapping segments[2]	RMSD, Å [3]	% idenity[4]	Score[5]	%AF2[6]	%PDB[7]
TREM2_HUMAN	6XDS	A	130	111-114:144-153:334:337-338:365-415:424-427:432-487:490-491	2.7	81	90	57	27
TRGC1_HUMAN	4LFH	G	112	113-219:222-223:225:227-228	1.4	95	92	65	50
TRGC2_HUMAN	1HXM	B	106	127-232	1.4	96	94	56	46
TRML1_HUMAN	2FRG	P	103	19:21-67:69:74-75:77-128	2.1	96	94	33	97
TUTLA_HUMAN	1V5J	A	99	625-723	1.6	96	86	8	92
TYOBP_HUMAN	2L35	A	54	6-12:14-27:29-30:32:35:46-72:74-75	3.3	13	75	48	86
TYRO3_HUMAN	1RHF	A	173	49-69:71-200:202-222:253	2.1	99	86	19	99
TYRP1_HUMAN	5M8T	A	446	25-470	0.3	99	98	83	100
UB2J2_HUMAN	2F4W	B	152	11-95:105-171	0.4	100	97	59	100
UBP19_HUMAN	1WH0	A	119	276:281:284-396:514-515:579-580	2.3	96	85	9	89
UBP30_HUMAN	5OHP	A	315	64-104:113-181:216-290:302-358:430-502	0.9	96	93	61	100
UCRI_HUMAN	5XTI	AB	49	159-161:163-183:186-187:189-196:199:254-256:258-259:261-269	3.7	4	94	18	86
UD2B7_HUMAN	2O6L	B	160	282-291:293-310:316-389:391-405:407-419:421-442:444-451	0.6	98	97	30	100
UDB15_HUMAN	6IPB	A	170	280-281:283-311:313-316:318-452	1.3	96	98	32	99
UFO_HUMAN	5U6B	B	287	520-561:564-609:611:615-702:704-709:711:713-814:816	1.3	98	85	32	99
UNC5A_HUMAN	4V2A	A	248	43-289:292	1.5	99	91	29	100
UXS1_HUMAN	4GLL	A	305	88-343:345-351:353-356:359-396	2.3	96	96	73	99
VAMP7_HUMAN	2DMW	A	121	8-127:169	1.6	97	92	55	92
VAMP8_HUMAN	7BV6	A	66	9-74	1	100	94	66	100
VAPA_HUMAN	2RR3	A	127	10-136	1.7	98	93	51	99
VAPB_HUMAN	3IKK	A	124	1-96:98-101:103-126	1	97	91	51	99

Table 1. Verification of AF2-generated models of 788 bitopic proteins from *Homo sapiens* by comparison with experimental structures from PDB (continuation).

UniProt ID	PDB ID[1]	Chain	N_{over}[2]	Overlapping segments[2]	RMSD, Å [3]	% idenity[4]	Score[5]	%AF2[6]	%PDB[7]
VAS1_HUMAN	6WM4	U	177	255-423:426-427:430-431:436-437:442:449	2.2	96	89	38	87
VCAM1_HUMAN	1VCA	A	199	25-222:250	1.6	100	89	27	100
VGFR1_HUMAN	5T89	Y	588	30-35:38-44:48-58:61-68:70-74:78-81:96-104:106:109-115:117-139:142-279:281-282:284-476:478-482:489:495-511:513-650:652-662:664-665	4.5	81	86	44	98
VGFR2_HUMAN	6GQO	A	308	805-808:813:816-944:955:995:997-1168	1.7	96	81	23	100
VGFR3_HUMAN	4BSJ	A	213	328-468:482:484-554	1.9	98	85	16	100
VISTA_HUMAN	6OIL	A	148	33-60:64-183	0.6	99	90	48	100
VRK2_HUMAN	5UU1	A	315	14-327:330	1	100	95	62	100
VSIG4_HUMAN	5IML	A	119	19-137	0.7	100	95	30	100
VTCN1_HUMAN	4GOS	A	115	35-149	0.8	99	97	41	100
VTI1B_HUMAN	2V8S	V	91	2-3:6-7:10-96	0.8	94	83	39	98
X3CL1_HUMAN	1F2L	D	70	28:30-98	1.5	99	91	18	100
XYLT1_HUMAN	6EJ7	A	706	252-730:732-733:735-959	0.5	100	96	74	100

[1] One PDB entry with the largest number of overlapped residues was selected for each protein.

[2] Overlapped segments of AF2 models and PDB structures; residues were numbered as in the AF2 model file.

[3] Sequence identity of overlapped residues in the model and the corresponding experimental structure after their superposition. The sequence-independent superposition option of TM-align was used.

[4] Root-mean-square deviation of C^{α}-atoms.

[5] Average AF2 confidence score (pLDDT) for overlapping residues.

[6] Percentage of the AF2 model that are overlapping with the PDB structure, $(N_{over}/N_{AF2})*100$, where N_{AF2} is the number of residues in the full-length AF2 model

[7] Percentage of PDB structure covered by overlapping residues, $(N_{over}/N_{PDB})*100$, where N_{PDB} is the number of residues in the PDB structure.

Same footnotes are used for Tables 2-5

UniProt ID	PDB ID	Chain	N_{over}	Overlapping segments	RMSD, Å	% Idenity	Score	% AF2	% PDB
AMPD_ARATH	2A3L	A	599	223-225:227-242:244-250:252-280:292-295:297-457:473-481:483:488-856	1.7	94	93	71	97
ANX1_ARATH	6FIG	A	386	25:27-411	0.6	100	93	45	100
ANX2_ARATH	5Y92	A	387	28-414	0.5	100	93	45	100
ARC6_ARATH	5D9R	A	132	668-799	0.6	100	91	16	100
BAK1_ARATH	3TL8	D	306	274-323:325-445:447-448:451-583	1	100	89	50	100
BRI1_ARATH	3RGZ	A	745	30-774	0.8	100	94	62	100
BRL1_ARATH	4J0M	A	724	31-60:64-757	0.7	100	93	62	100
CERK1_ARATH	4EBZ	A	200	25-224	0.7	100	91	32	100
CRL_ARATH	6LIX	B	196	42-237	0.9	100	97	73	100
ERL1_ARATH	5XJX	K	544	28-571	0.9	100	95	56	100
ERL2_ARATH	5XKN	A	540	29:31-566:568-570	1.2	99	95	56	100
FERON_ARATH	6A5B	A	376	34-65:70-201:207-209:216-424	0.4	100	93	42	100
FKB42_ARATH	2IF4	A	258	35-292	0.9	100	96	71	100
FLS2_ARATH	4MN8	A	759	26-784	1.2	100	96	65	100
FLU_ARATH	5CHE	E	119	196:198-315	0.6	99	96	38	100
FUT1_ARATH	5KWK	B	457	95-399:407-558	0.4	100	96	82	100
GLDH_ARATH	7A24	z	436	12-16:124-125:135:138-143:145-350:352-355:367-369:393-399:406:433-447:451:455-457:459-472:477-506:508-510:513-528:531:533-549:562-580:582:584:586-598:600:602-603:605-668	3.6	78	96	71	87
GSO1_ARATH	6S6Q	A	833	25-38:40-42:44-859	1.7	98	96	67	100
HAP2_ARATH	5OW3	C	445	25-75:86:88-142:145:147-263:275-494	1.3	99	92	63	100
HXK1_ARATH	4QS7	A	455	34-424:430-492:496	0.8	100	96	92	100

Table 2. Verification of AF2-generated models of 52 bitopic proteins from *Arabidopsis thaliana* by comparison with experimental structures from PDB (continuation).

UniProt ID	PDB ID	Chain	N_{over}	Overlapping segments	RMSD, Å	% Idenity	Score	% AF2	% PDB
NCPR2_ARATH	5GXU	A	532	104-140:145-147:154-163:167-173:180-188:190-191:193-195:198-205:207-208:211:213-219:223-240:242-243:245-247:249-255:258-259:261-263:265-280:324-400:416:418-731	3.8	80	94	75	96
NDADA_ARATH	7A24	Z	123	17-20:35:38:40-135:137-140:142-152:154-159	2.8	88	94	86	88
NDUA1_ARATH	7A24	V	57	2-57:59	0.8	98	92	88	100
OE64M_ARATH	6Q3Q	A	121	264-265:268:486-603	0.9	97	95	20	100
P2C57_ARATH	4YZH	A	294	58-349:351-352	1.1	99	96	76	100
P2C70_ARATH	1MZK	A	120	181-299:302	1.4	98	88	21	98
PDLP5_ARATH	6GRE	B	207	29-235	0.9	100	95	69	100
PDLP8_ARATH	6GRF	B	211	26-236	0.3	100	97	76	100
PDV1_ARATH	5U9O	E	46	1-24:27:29-30:49-50:54-55:59:162-163:166-168:170-171:174:177-178:181-182:316-317	4.5	9	88	17	32
PEPR1_ARATH	5GR8	A	710	29-733:735-738:740	1.9	99	96	63	100
PRK3_ARATH	5WLS	A	208	25-35:37-233	0.7	95	93	33	100
PRK6_ARATH	5YAH	B	215	27-241	1.5	100	91	33	100
PSBE_ARATH	5MDX	E	71	10-54:56-58:60-82	1.4	96	95	86	99
PSBH_ARATH	5MDX	H	52	13:15-64:66	1.7	96	97	71	100
PSKR1_ARATH	4Z63	A	610	30-634:637-641	1	99	94	61	100
RGI3_ARATH	5HZ3	B	623	56:58-84:92-686	1.2	99	94	57	100
RLK5_ARATH	5IYX	A	596	21-616	1	100	95	60	100
SBIR1_ARATH	6R1H	A	184	34-56:64-220:222-225	1.3	98	89	29	100
SCO11_ARATH	6N5U	A	161	172-332	0.7	100	94	48	100
SERK1_ARATH	6FG8	A	188	25-212	0.8	100	89	30	100
SERK2_ARATH	6G3W	A	185	30-213:215	0.5	100	91	29	100

Table 2. Verification of AF2-generated models of 52 bitopic proteins from *Arabidopsis thaliana* by comparison with experimental structures from PDB (continuation).

UniProt ID	PDB ID	Chain	N_{over}	Overlapping segments	RMSD, Å	% Idenity	Score	% AF2	% PDB
TBL29_ARATH	6CCI	A	354	133-486	0.9	100	95	73	100
TDR_ARATH	5GIJ	B	599	31-59:61:65-633	1.1	99	95	58	99
TIC40_ARATH	2LNM	A	60	385-387:389-445	1.6	97	77	13	97
TMK1_ARATH	4HQ1	A	424	24-447	0.3	100	97	45	100
TMK3_ARATH	7BRC	A	425	25:27-449:451	0.8	100	95	45	100
TMM_ARATH	5XJO	C	367	52-416:421:425	0.5	99	96	74	100
TO203_ARATH	1ZU2	A	145	5:7-150	2	99	93	72	92
U603_ARATH	3PW9	A	153	83-235	0.7	99	86	54	100
VSR1_ARATH	4TJV	A	155	25-28:30-130:133-134:137-140:142:146-184:186-189	2.2	92	92	25	94
XXT1_ARATH	6BSW	A	337	116-452	0.2	100	97	73	100
Y1719_ARATH	6G3W	B	189	25-213	1	100	91	31	100

Table 3. Verification of AF2-generated models of 69 bitopic proteins from *Saccharomyces cerevisiae* by comparison with experimental structures from PDB.

UniProt ID	PDB ID	Chain	N_{over}	Overlapping segments	RMSD, Å	% Idenity	Score	% AF2	% PDB
ATG32_YEAST	5WLP	A	144	197-340	2	98	84	27	99
ATP18_YEAST	6B8H	i	59	1-59	1.1	100	95	100	100
ATPK_YEAST	6WTD	U	54	50:53-62:64-106	2.5	80	90	53	79
COX13_YEAST	6T15	k	118	10:12-128	1.7	99	95	91	100
COX5A_YEAST	6HU9	e	133	21-153	0.9	100	94	87	100
COX5B_YEAST	6T15	e	134	18-151	1.2	100	94	89	100
COX7_YEAST	6T15	g	59	2-60	1.1	100	96	98	100
COX8_YEAST	6YMX	h	51	28-78	1.5	100	95	65	100
COX9_YEAST	6T15	i	50	12-60:62	1.9	98	98	85	91
CP51_YEAST	5EQB	A	525	6-530	0.7	100	98	99	99
CUE1_YEAST	2MYX	A	70	50-52:54-120	1.9	96	82	34	93
CWH41_YEAST	4J5T	A	780	31-258:265-506:508-509:526-833	0.9	99	94	94	99
CYB2_YEAST	1KBI	A	504	81-380:387-388:390-591	0.5	100	94	85	100
EMC1_YEAST	7KRA	A	694	25-96:99:101-201:206-230:249-250:252-268:270:272-285:287-410:423-755:757-760	2.7	95	84	91	100
EMP46_YEAST	2A6X	B	219	53:55-272	0.3	99	92	49	100
EMP47_YEAST	2A6Y	A	224	36-257:259-260	0.7	98	94	50	97
ERV2_YEAST	1JRA	A	106	74-179	1.4	100	96	54	100
FET3_YEAST	1ZPU	A	529	22-531:533-540:542-543:545-553	0.4	97	98	83	100
FIS1_YEAST	1Y8M	A	135	7-134:136-138:140:147:150-151	2.3	96	92	87	94
GLRX6_YEAST	5J3R	A	114	112-225	0.2	100	98	49	100
HRD3_YEAST	5V7V	A	613	26-257:259-264:266-270:316:318-685:687	1.5	98	92	74	100
IRE1_YEAST	3SDJ	D	420	663-759:761-771:773-840:843-844:846-847:849-852:854-867:896-1117	1.2	96	88	38	100
KEX1_YEAST	1AC5	A	483	23-505	0.3	100	96	66	100
KEX2_YEAST	1R64	A	481	121-601	0.3	100	97	59	100

Table 3. Verification of AF2-generated models of 69 bitopic proteins from *Saccharomyces cerevisiae* by comparison with experimental structures from PDB (continuation).

UniProt ID	PDB ID	Chain	N_{over}	Overlapping segments	RMSD, Å	% Idenity	Score	% AF2	% PDB
KRE2_YEAST	1S4N	A	337	104-114:116:118-442	0.2	100	98	76	100
KTR4_YEAST	5A07	A	395	70-464	0.2	100	98	85	100
LAM4_YEAST	6BYM	A	197	946-1142	1	100	91	15	98
LAM6_YEAST	5YQR	A	112	312:314-424	1.7	96	86	16	42
MDM38_YEAST	3SKQ	A	227	182-408	0.3	100	92	40	100
MNN9_YEAST	3ZF8	A	288	95-189:202-394	0.7	100	93	73	100
MNS1_YEAST	1G6I	A	510	34-365:371-409:411-549	0.3	99	98	93	100
MSP1_YEAST	5W0T	A	196	50-111:114-140:142-158:160-162:164-176:178-199:206-216:218-229:231-259	1.4	100	88	54	68
NCPR_YEAST	2BF4	A	645	47-691	0.4	100	96	93	100
NYV1_YEAST	2FZ0	A	149	1-149	2.6	99	82	59	100
OST1_YEAST	6EZN	A	438	25-98:111-474	1.2	100	90	92	100
OSTB_YEAST	6C26	B	394	24-388:390:393-394:396-414:417-418:423-427	2.4	94	90	92	99
PEX13_YEAST	1JQQ	B	78	293-294:297:301-307:309-376	2.2	87	85	20	95
PEX15_YEAST	5VXV	A	211	43-64:66-80:82-92:94-166:168-177:179-210:212-259	0.9	97	95	55	100
PEX22_YEAST	2Y9P	B	122	57-147:152-182	1.1	100	90	68	98
QCR10_YEAST	6HU9	U	54	36:43:45-52:54-90:92-93:95-99	3.2	82	75	70	71
QCR8_YEAST	6YMX	H	88	12-99	1.6	100	91	94	95
QCR9_YEAST	6T15	T	57	1-2:4-58	1.8	96	95	86	100
SC61G_YEAST	2WWA	B	60	21-80	2.1	100	91	75	100
SCO1_YEAST	2B7J	B	173	108:114-283:285-286	1.6	98	89	59	96
SCS2_YEAST	6LP4	A	126	3-128	0.7	99	97	52	100
SEC12_YEAST	6X90	B	344	1-344	0.7	100	97	73	100
SEC66_YEAST	6ND1	E	139	29-80:82:91-157:159:162-169:171-180	1.7	86	94	67	100
SNC1_YEAST	3B5N	A	61	26-86	0.7	98	91	52	100

Table 3. Verification of AF2-generated models of 69 bitopic proteins from *Saccharomyces cerevisiae* by comparison with experimental structures from PDB (continuation).

UniProt ID	PDB ID	Chain	N_{over}	Overlapping segments	RMSD, Å	% Idenity	Score	% AF2	% PDB
SOP4_YEAST	7KTX	G	143	20-31:33-40:42-127:129-140:142-159:163-169	2	82	86	61	95
SRPB_YEAST	1NRJ	B	191	36-188:203-206:211-244	0.6	100	94	78	100
SSO1_YEAST	1FIO	A	188	30-166:168-179:184:186-221:223-224	1.8	93	87	65	99
SSO2_YEAST	5LG4	A	187	33-169:171-177:179:182-183:188-190:192-226:228-229	1.9	91	88	63	99
TIM14_YEAST	2GUZ	A	71	98-168	0.9	99	90	42	100
TIM21_YEAST	6K7D	A	147	141-148:152-154:232-233:235-242:244-248:444-446:448-496:503-570:572	3.9	76	90	62	29
TIM50_YEAST	4QQF	A	191	167-171:176-179:183-362:364-365	2.3	95	92	40	96
TIM54_YEAST	6LO8	B	175	36-62:64-80:82-150:153:240-264:371-378:385-386:388-413	0.9	85	94	37	99
TLG1_YEAST	2C5I	T	93	6-61:63-99	1.2	100	81	42	100
TOM22_YEAST	6UCV	J	55	77-131	1.2	9	95	36	100
TOM70_YEAST	2GW1	A	395	88-97:100-101:107-131:342-698:701	2.2	91	88	64	81
TOM71_YEAST	3FP3	A	498	107:109-120:122-138:140:146-254:261-534:544-627	1.2	97	93	78	100
UCRI_YEAST	6T0B	E	185	31-215	2.7	100	94	86	100
UPPS_YEAST	6JCN	A	206	151-248:261-262:264-369	1.5	98	92	55	100
USA1_YEAST	6VK0	D	64	12-15:28-29:38:41-44:47:75-77:80:88-102:165-166:170-173:197:207-211:213-219:221-222:225-226:228-231:256:266-270	4.9	8	78	8	66
VAM3_YEAST	1HS7	A	97	23-119	1.3	100	80	34	100
VOA1_YEAST	6PE4	B	47	212:214:216-250:252-261	3.1	79	71	18	89
VPS64_YEAST	6A8W	A	128	159-166:168-190:198-268:270-295	0.7	99	89	21	100
VTI1_YEAST	3ONJ	A	97	3-98:101	1	100	82	45	100

Table 3. Verification of AF2-generated models of 69 bitopic proteins from *Saccharomyces cerevisiae* by comparison with experimental structures from PDB (continuation).

UniProt ID	PDB ID	Chain	N_{over}	Overlapping segments	RMSD, Å	% Idenity	Score	% AF2	% PDB
YME1_YEAST	2MV3	A	87	98-181:183-185	1.7	92	85	12	99
YSP2_YEAST	5YQI	A	166	847-850:852-912:916-1015:1018	1.3	98	92	12	100

Table 4. Verification of AF2-generated models of 34 bitopic proteins from *Escherichia coli* by comparison with experimental structures from PDB.

UniProt ID	PDB ID	Chain	N_{over}	Overlapping segments	RMSD, Å	% Idenity	Score	% AF2	% PDB
ATPF_ECOLI	5T4P	I	136	2-129:131-135:140:155-156	3.1	93	94	87	88
BCSB_ECOLI	7LBY	B	650	68-78:80-85:88-449:451-459:475-609:623-633:652-653:672-754:757:759-785:788:791-792	2.6	90	91	83	98
CADC_ECOLI	3LY9	A	322	190-511	1.3	100	93	63	100
CCME_ECOLI	1SR3	A	98	38-135	1.6	100	90	62	86
DAMX_ECOLI	2LFV	A	90	103-106:359-444	2.5	96	88	21	85
DEGS_ECOLI	1TE0	A	307	37-180:183-223:232-275:288-365	2.6	99	90	86	97
DSBE_ECOLI	3K8N	A	153	30-33:35-182:184	1.8	98	94	83	100
EXBD_ECOLI	2PFU	A	75	79-153	1.8	100	87	53	76
FDNH_ECOLI	1KQF	B	284	2-285	1.6	100	94	97	98
FTSB_ECOLI	4IFF	C	65	25-38:42-91:93	2.2	8	84	63	79
FTSH_ECOLI	1LV7	A	248	138-143:146-147:149-177:183-221:225-227:231-398:443	2.5	95	87	39	99
FTSI_ECOLI	6HZQ	A	301	234-280:293:296-297:301-336:339-391:401-402:408-567	1.1	99	96	51	100
FTSN_ECOLI	1UTA	A	72	242-245:247-291:296-304:306:308-311:316-324	2.9	76	91	23	94
FTSQ_ECOLI	5Z2W	A	205	61-265	1.7	100	92	74	98
GSPH_ECOLI	2KNQ	A	136	31-32:34-84:89-171	2.2	98	83	80	96
KDPC_ECOLI	7BH2	C	190	1-190	1	100	95	100	100
LPTC_ECOLI	4B54	A	125	53:59-182	0.8	98	97	65	100

Table 4. Verification of AF2-generated models of 34 bitopic proteins from *Escherichia coli* by comparison with experimental structures from PDB (continuation).

UniProt ID	PDB ID	Chain	N_{over}	Overlapping segments	RMSD, Å	% Idenity	Score	% AF2	% PDB
MACA_ECOLI	5NIL	F	335	32-320:322-331:333:335-347:349-352:354-365:367-370:373:376	2.8	89	93	90	99
MBHS_ECOLI	4GD3	S	293	6:50-341	0.5	99	95	79	96
MBHT_ECOLI	6SZK	SSS	268	46-313	0.3	100	97	72	100
MLAD_ECOLI	7CGN	J	143	1-2:4-15:17-26:28-29:35:37:39-153	3.2	82	94	78	99
MRDA_ECOLI	6G9F	A	539	57-549:568-613	0.7	100	96	85	100
PBPB_ECOLI	5HLB	A	731	66-67:69-234:236-237:239-257:260-792:794-802	1.1	96	94	87	100
PPID_ECOLI	2KGJ	A	98	264-360:369	1.9	97	93	16	96
PQIB_ECOLI	5UVN	B	392	41-42:44-347:355-387:401:403-431:433:436-452:455-456:461:494:498	2.5	93	86	72	94
RSEA_ECOLI	1OR7	C	66	1-66	1.2	100	92	31	100
SPPA_ECOLI	3BF0	C	478	56-72:79:90:93-539:541-552	0.7	97	96	77	100
TAMB_ECOLI	5VTG	A	131	974-975:977-995:1024:1026-1106:1108:1111-1114:1118-1140	2	94	90	10	96
TATA_ECOLI	2LZS	B	48	1-3:5-42:44-46:48-49:51:53	2.4	83	88	54	100
TOLA_ECOLI	3QDR	A	92	328:330-350:352-421	1.2	99	90	22	100
TOLR_ECOLI	5BY4	A	105	37-141	1.3	100	79	74	100
YCBB_ECOLI	6NTW	A	502	51-62:64-68:70:73-74:76-78:80-266:315-457:460-467:469-472:474-478:480-600:604:608-617	2.4	91	94	82	99
YEBT_ECOLI	6V0C	C	820	49:52-60:62-65:67-77:80-107:111-123:126-127:129:131-132:134-146:150-346:348-365:367-369:371-383:385-889	4.8	87	85	94	99
ZIPA_ECOLI	1F47	B	144	185-328	0.9	99	96	44	100

Table 5. Verification of AF2-generated models of 4 bitopic proteins from *Methanococcus jannaschii* by comparison with experimental structures from PDB.

UniProt ID	PDB ID	Chain	N_{over}	Overlapping segments	RMSD, Å	% Idenity	Score	% AF2	% PDB
FLAB1_METJA	5YA6	A	159	59-217	0.4	100	92	73	98
MTRA_METJA	5L8X	A	170	3-172	0.2	100	95	71	99
SECE_METJA	2YXR	B	65	3-67	1.1	100	94	88	100
Y781_METJA	2JNQ	A	186	391-392:395-396:402-575:599-600:602:630-633:661	2.2	92	81	26	100

Table 6. AF2-generated models with coordinate R.M.S.D. larger than 5 Å from experimental structures

UniProt ID	PDB ID	Chain	N_{over}	Overlapping segments	RMSD, Å	% Idenity	Score	% AF2	% PDB
**Models compared with structures composed of multiple domains connected by flexible loops**									
BCS1_YEAST	6SH4	A	358	49-54:57-83:85-95:97-111:113:115-126:130:133-147:149-154:156-163:166-170:175-177:180-187:189-210:213-220:225-233:235-238:240-297:299:301-311:315-324:326-327:333-343:345-346:359-371:376-387:390-391:393-467	5.5	59	86	79	95
INSR_HUMAN	4ZXB	E	623	110-115:117:120-121:123-128:133-145:147-150:152-171:174-181:184-186:235-236:412-415:427-435:440-459:462-468:470-703:718:720-844:881:970-1126	5.1	84	88	45	78
LY75_HUMAN	7JPT	A	915	29:31-86:92:94-136:138-143:145-492:497:499-760:775-812:861:966-984:1010-1015:1020-1028:1031-1032:1035-1041:1108-1112:1131-1142:1182-1202:1231-1245:1248-1253:1266-1269:1299-1301:1304-1315:1359-1363:1401-1402:1419-1425:1461-1469:1562:1567-1568:1957-1963:2051-2052:2054-2055	5.2	79	79	53	69
PCD10_HUMAN	6VG4	A	633	19-29:33-41:46-49:54-55:59:62-70:72-109:114-219:236-237:239-592:594-607:609-657:659-663:665-693	5.3	92	93	61	98

VLDLR_HUMAN	6BYV	A	102	456-460:470-476:491-493:500-506:528-531:533-537:543-549:573-591:596-598:610:621-624:627:629-632:634:637-642:645:652-661:685:688-689:692-696:698-702:704	5.1	7	93	12	84
**Models compared with PDB entries representing proteins with hybrid sequences**									
ATG40_YEAST	7BRN	A	65	21-26:28-29:32-34:36:38-46:48-49:51-55:58:63-64:67:69-70:73:76-77:79-80:83:113:116:119-120:123:125-127:130:205-215:228:275:278-279:294	5.2	9	69	25	49
HG2A_HUMAN	4AH2	B	83	69:72-74:76-78:80-82:106:109-110:113-114:126:130:133:246-259:261:263:265:267-269:273-274:282-285:288:290:292:295:305-308:310-320:338-342:355-363:365-370	6.3	7	85	28	42
MIA40_YEAST	2ZXT	A	121	212-213:216-225:333-337:449-453:472-474:484-488:555-565:596-598:601:608-679:681-683:686	5.2	58	74	30	27
RENR_HUMAN	3LBS	B	157	28-33:36-37:40:51-57:59-62:64-68:77-81:83:86:88:94:98-102:126-133:135:137-144:146-147:150-154:163-166:171:174:179-180:183-185:190:194:199-216:239:270-275:277:308:310-321:324-326:329-330:341-352:378:400:404-410:412-415:453:456-457:460-462:487-492	6.8	6	85	45	42
SEM4C_HUMAN	6N5Z	A	90	214:240:253:296-321:328-350:358-362:373-377:463-465:467:469-479:499:505:507-511:517-522	5.2	6	96	11	57
TNR25_HUMAN	5YGP	D	145	32-40:45:48:50-55:63:87:89-95:117-119:122-124:126-134:141-150:197-234:238-239:462:465-490:500-513:524:528-529:556:559-567	7.6	6	88	35	32
